# A fully synthetic self-adjuvanting globo H-Based vaccine elicited strong T cell-mediated antitumor immunity†
†Electronic supplementary information (ESI) available: Synthetic procedure for **6**; NMR spectra of **1**, **6**, and **7**, and additional immunological data. See DOI: 10.1039/c5sc01402f



**DOI:** 10.1039/c5sc01402f

**Published:** 2015-09-22

**Authors:** Zhifang Zhou, Guochao Liao, Satadru S. Mandal, Sharad Suryawanshi, Zhongwu Guo

**Affiliations:** a Department of Chemistry , Wayne State University , 1501 Cass Avenue , Detroit , Michigan 48202 , USA . Email: zwguo@chem.wayne.edu ; Tel: +1-313-577-2557

## Abstract

Therapeutic cancer vaccines based on the abnormal glycans expressed on cancer cells, such as the globo H antigen, have witnessed great progress in recent years. For example, the keyhole limpet hemocyanin (KLH) conjugate of globo H has been on clinical trials as a cancer vaccine. However, such vaccines have intrinsic problems, such as inconsistence in eliciting T cell-mediated immunity in cancer patients and difficult quality control. To address the issue, a structurally defined fully synthetic glycoconjugate vaccine composed of globo H and monophosphoryl lipid A (MPLA) was developed. The new vaccine was shown to elicit robust IgG1 antibody responses and T cell-dependent immunity, which is desired for anticancer vaccines, and induce significantly faster and stronger immune responses than the globo H–KLH conjugate. Moreover, it was self-adjuvanting, namely, inducing immune responses without the use of an external adjuvant, thus MPLA was not only a vaccine carrier but also a build-in adjuvant. It was also found that antibodies induced by the new vaccine could selectively bind to and mediate strong complement-dependent cytotoxicity to globo H-expressing MCF-7 cancer cell. All of the results have demonstrated that the globo H–MPLA conjugate is a better cancer vaccine than the globo H–KLH conjugate under experimental conditions and is worth further investigation and development.

## Abstract 2

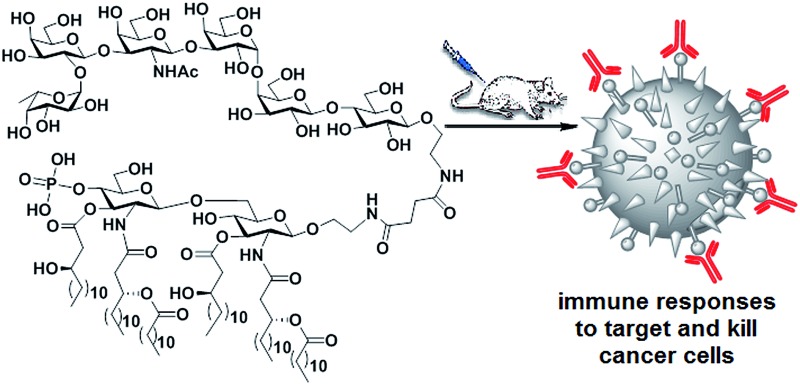
Fully synthetic, self-adjuvanting monophosphoryl lipid A–globo H conjugate elicited strong T cell-mediated immunity that could target and kill breast cancer.

## Introduction

The abnormal glycans expressed by cancer cells, known as tumor-associated carbohydrate antigens (TACAs), are useful epitopes for the development of therapeutic cancer vaccines,^1,2^ as they are abundant and exposed on the cancer cell surface and thereby easy targets for the human immune system. Among many TACAs identified so far,^3,4^ the globo H antigen, which is a rather tumor-specific hexasaccharide antigen, is especially attractive. Globo H was first discovered in conjugation with lipids on human breast cancer cell MCF-7,^5,6^ and later on was also found on a variety of other epithelial tumors, such as lung, colon, ovarian, and prostate cancer.^7^ As a result, globo H-based anticancer vaccines can be broadly useful for treating different tumors, and globo H has become a hot topic for synthetic studies and cancer vaccine design.^8,9^

However, similar to most carbohydrate antigens, globo H itself is poorly immunogenic and T cell-independent,^10,11^ while T cell-mediated immunity, which means antibody affinity maturation and improved immunological memorization and cytotoxicity to cancer cells compared to purely humoral or antibody-mediated immunity, is critical for cancer immunotherapy.^12^ The conventional method to deal with the issue is to couple carbohydrate antigens with an immunologically active carrier protein to form conjugate vaccines, a strategy that not only increases the immunogenicity of carbohydrates but also switches them from T cell-independent to T cell-dependent antigens.^12^ The most commonly used carrier protein in the development of anticancer vaccines is keyhole limpet hemocyanin (KLH).^13^ The KLH conjugates of globo H have made great progress as therapeutic cancer vaccines. For example, used together with an external adjuvant such as QS-21, they have been shown to elicit strong immune responses and thus have been in phase III clinical trials for the treatment of breast and prostate cancer,^8,14^ demonstrating the great potential of globo H-based vaccines for cancer immunotherapy.

Despite that the KLH conjugates of globo H as anticancer vaccines have shown promising results,^15^ there are still issues in their clinical application. The KLH–globo H conjugates usually provoked high levels of antigen-specific IgM antibodies, but the levels of IgG antibodies, which indicate T cell-mediated immunity, were relatively low in patients.^8^ This was probably due to the fact that the carrier protein itself could elicit strong immunity and thereby suppress the immune response to the carbohydrate antigen.^16,17^ Furthermore, due to the multivalent property of carrier proteins and the unpredictability of the conjugation reaction, it is difficult to control the coupling sites and the loading levels of carbohydrates in TACA–protein conjugates, causing problems in their quality control. In addition, traditional vaccines have to be used with an external adjuvant to be effective, which can lead to serious side effects.^8^ For example, inflammatory responses at the injection site and systemic syndromes such as fever, arthralgias, and myalgias induced by QS21 have been reported during the clinical trials of KLH–globo H conjugates.^18–20^ To overcome these problems associated with protein–TACA conjugates, a variety of new constructs of carbohydrate-based vaccines using non-protein carriers, *e.g.*, dendrimers,^21,22^ polysaccharides,^23^ nanoparticles,^24^ and lipids,^25–31^ and vaccines with self-adjuvanting properties^32–39^ have been designed and explored. Among them, lipid carrier-based fully synthetic glycoconjugate vaccines are particularly attractive as they possess homogeneous and defined chemical structures, which would not only streamline their characterization and quality control but also enable detailed immunological studies to gain insights into their functions, functional mechanisms, and structure-activity relationships to guide the design and further optimization of related vaccines.

We have recently explored a new class of carrier molecules, namely, 1-*O*-dephosphorylated monophosphoryl derivatives of lipid A, which can be used for the development of fully synthetic glycoconjugate vaccines. Lipid A is the core hydrophobic domain of bacterial lipopolysaccharides (LPSs) and mainly responsible for the immunostimulatory activity of LPSs.^40^ Its monophosphoryl derivatives, known as monophosphoryl lipid A (MPLA), also have very strong immunostimulatory activity. They act through interaction with toll-like receptor 4 (TLR4) to stimulate a downstream signaling cascade and eventually the production of cytokines and chemokines,^41–44^ such as tumor necrosis factor-α (TNF-α), interleukin-1β (IL-1β), IL-6, interferon-β (IFN-β), *etc.* ^44,45^ Different from lipid A, however, MPLA is essentially nontoxic, and therefore has been recently approved for clinical use as a human vaccine adjuvant.^46^ We have demonstrated that the MPLA conjugates of artificial TACA analogs could elicit robust immune responses in the absence of an external adjuvant,^26,28^ suggesting the potential of creating fully synthetic, self-adjuvanting glycoconjugate vaccines with MPLA as a carrier molecule.

**Fig. 1:**
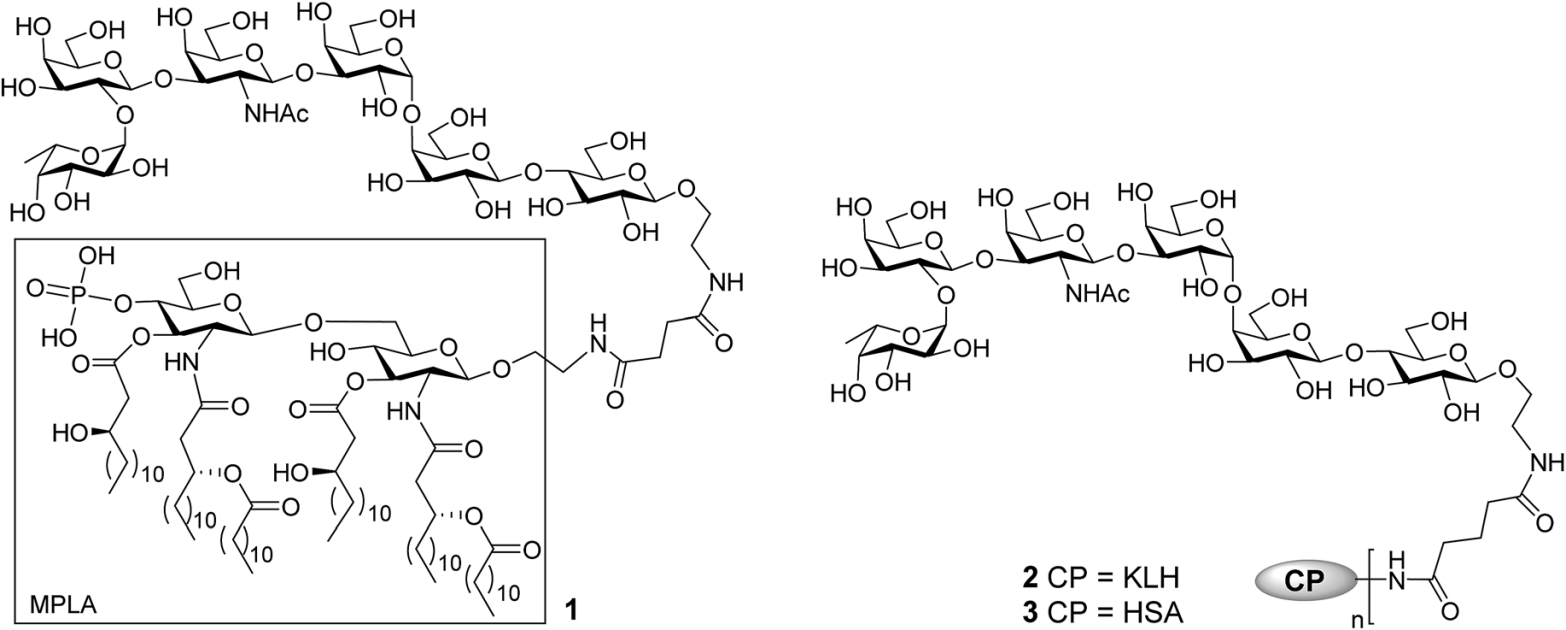
The structure of MPLA–, KLH–, and HSA–globo H conjugates **1**, **2**, and **3**.

The current work aimed at developing fully synthetic anticancer vaccines based on globo H. For this purpose, globo H was coupled with the synthetic monophosphoryl derivative of *Neisseria meningitides* lipid A – an optimized carrier molecule.^28^ The resultant glycoconjugate **1** (Fig. 1) was immunologically evaluated in mice. Its results were compared with that of the KLH–globo H conjugate **2** that was on clinical trial. In the meantime, the human serum albumin (HSA)–globo H conjugate **3** was also prepared and used as the coating antigen for enzyme-linked immunosorbent assays (ELISA) of globo H-specific antibodies.

## Results and discussion

### Preparation of glycoconjugates **1–3**

**Scheme 1:**
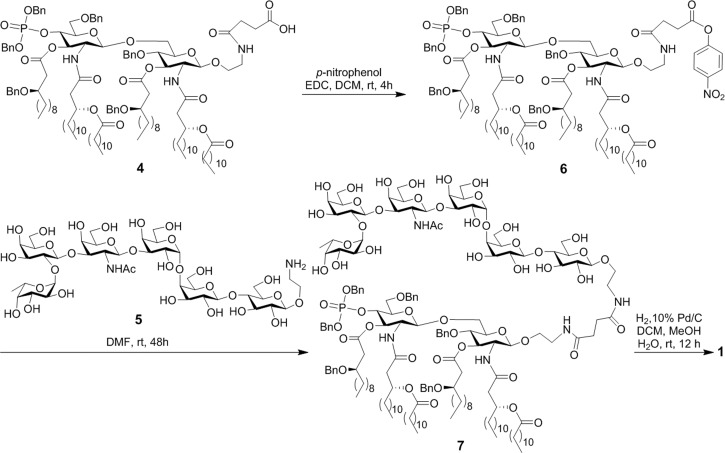
Synthesis of the MPLA–globo H conjugate **1**.

The MPLA–globo H conjugate **1** was prepared by coupling a carboxylic acid derivative of *N. meningitidis* MPLA (**4**) with a derivative of globo H (**5**) that had a free amino group attached to its reducing end, according to the procedure outlined in Scheme 1. The chemical syntheses of **4** and **5** utilized here were described previously.^28,47,48^ Therefore, **4** was converted into an activated ester **6** by reacting with *p*-nitrophenol and 1-ethyl-3-(3-dimethylaminopropyl)carbodiimide (EDC) hydrochloride.^28^ The activated ester **6** was then subjected to a regioselective reaction with **5** to afford the protected MPLA–globo H conjugate **7**. Finally, all of the benzyl (Bn) groups in **7** were removed through hydrogenolysis under an H_2_ atmosphere using 10% Pd/C as the catalyst to produce the desired MPLA–globo H conjugate **1** in a good overall yield (34%). The synthetic target and all of the intermediates were characterized with NMR and MS spectrometry.

**Scheme 2:**
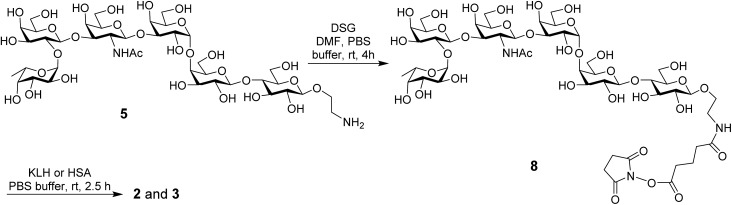
Synthesis of the protein–globo H conjugates **2** and **3**.

The KLH and HSA conjugates of globo H were readily prepared by coupling **5** with KLH and HSA through a bifunctional glutaryl linker (Scheme 2). Here, the glutaryl linker was selected not only because it provided reliable conjugation reactions but also because this simple linker did not induce immune responses or have a negative impact on the immunological properties of the resulting glycoconjugates.^49^ Treatment of **5** with a large excess (15 eq.) of disuccinimidal glutarate (DSG) in DMF produced activated ester **8**, which reacted with KLH or HSA in 0.1 M PBS buffer to afford glycoconjugates **2** and **3**. In these conjugates, one of the acyl groups of the glutaryl linker was attached to the free amino group in **5** and the other acyl group was attached to the free amino groups of proteins. After purification with a Biogel A 0.5 column, **2** and **3** were analyzed by the phenol-sulfuric acid method,^50^ with corresponding protein as control, to assess their carbohydrate loadings, which were 8% and 14%, respectively. The results showed that the coupling reactions were effective and the antigen loading levels were in the desired range (5–20%) for glycoconjugate vaccines or capture reagents used in ELISA.^51^ In addition, the HSA conjugate **3** was also analyzed with MALDI-TOF MS to obtain similar result (12%, ESI†), indicating that the phenol-sulfuric acid method could be used confidently to estimate the carbohydrate loadings of glycoproteins. On the other hand, the KLH conjugate **2**, of which the molecular weight was too big for MS analysis, was studied with SDS-PAGE, and an obvious increase in molecular weight of the glycoconjugate as compared to that of the protein itself (ESI†) proved the successful conjugation between KLH and globo H as well.

### Immunological evaluation of the MPLA– and KLH–globo H conjugates **1** and **2**

These studies were carried out with female C57BL/6J mice. The MPLA conjugate **1** was administered in a liposomal formulation prepared with 1,2-distearoyl-*sn*-glycero-3-phosphocholine (DSPC) and cholesterol in a molar ratio of 10 : 65 : 50 by a reported method.^28^ By incorporating conjugate **1** in liposomes, we anticipated to improve not only its solubility to get a homogeneous formulation of **1** but also its immunogenicity.^52,53^ Our previous studies^26^ have already disclosed that an external adjuvant would not improve the immunogenicity of MPLA conjugates but might have a negative impact, thus conjugate **1** was used without an external adjuvant. On the other hand, as the KLH conjugate **2** would be ineffective without an adjuvant, it was used as an emulsion with Freund's complete adjuvant (CFA) that is commonly used in animal study. In this case, **2** was first dissolved in PBS buffer and then thoroughly mixed with CFA before use.

For mouse immunization, **1** (16 μg of liposomal formulation containing 5.4 μg of Globo H) and **2** (38 μg of adjuvant emulsion containing 3.1 μg of Globo H) were individually administered to each group of six mice through subcutaneous (s.c.) injection. Our studies have showed that the dosage of glycoconjugate vaccines within the range of 1–9 μg of carbohydrates had little impact on the induced antibody titers. The immunization schedule included boosting each mouse three times on days 14, 21, and 28, respectively, by injection of the same vaccine preparations after the initial immunization on day 1. Blood samples were collected from each mouse on day 0 before the initial inoculation (blank controls) and on days 21, 27, and 38 after immunizations. The blood samples were used to prepare sera according to standard protocols. The sera were then analyzed by ELISA using HSA–globo H conjugate **3** as the capture reagent to coat plates. The titers of both total antibodies (anti-kappa) and various antibody isotypes, including IgG1, IgG2b, IgG2c, IgG3, and IgM antibodies, were assessed. Here, IgG2c antibody, instead of IgG2a, was analyzed since C57BL/6 mouse was found to express IgG2c antibody instead of the allelic IgG2a antibody.^54,55^ For the analysis of antibody titers, ELISA plates were coated first with conjugate **3** and then with a blocking buffer [1% bovine serum albumin (BSA) in PBS]. Thereafter, half-log serially diluted mouse sera from 1 : 300 to 1 : 656 100 in PBS were added to the plates. After incubation, the plates were washed and then incubated with 1 : 1000 diluted solutions of alkaline phosphatase (AP)-linked goat anti-mouse kappa, IgG1, IgG2b, IgG2c, IgG3, and IgM antibodies, respectively. Finally, the plates were developed with a *p*-nitrophenylphosphate (PNPP) solution, which was followed by colorimetric readout at 405 nm wavelength. Antibody titers were calculated from the curves obtained by drawing the adjusted optical density (OD) values, that is, after subtraction of the OD values of the blanks, against the serum dilution numbers and were defined as the serum dilution numbers yielding an OD value of 0.1.^56^

**Fig. 2:**
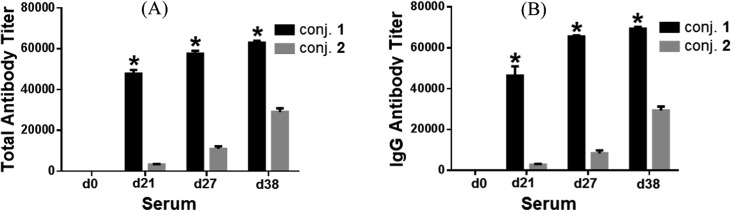
The overall total antibody (A) and total IgG antibody (B) titers of pooled day 0, 21, 27, and 38 sera derived from mice immunized with conjugates **1** and **2**. Antibody titers were defined as the serum dilution numbers yielding an OD value of 0.1, calculated from the curves obtained by drawing the OD values against the serum dilution numbers in the ELISA of mouse sera. The mean of antibody titers of three parallel experiments is shown for each sample, and the error bar shows the standard error of mean (SEM) of three replicate experiments. *Compared to the serum obtained on the same day after immunization with conjugate **2**, the difference in antibody titers is statistically significant (student's *t* test, *P* < 0.05).

Fig. 2 depicted the overall total antibody titers and total IgG antibody titers of the pooled day 0, 21, 27, and 38 sera derived from each group of mice inoculated with conjugates **1** and **2**, respectively. Clearly, the day 21 serum obtained from mice inoculated with the MPLA conjugate **1** twice on day 1 and day 14 already showed high globo H-specific total and IgG antibody titers (47 824 and 46 449, respectively, Fig. 2A and B), indicating that **1** could rapidly elicit robust immune responses. The anti-globo H antibody titers, especially the IgG antibody titers of the day 27 and 38 antisera (65 577 and 69 406, respectively), induced by **1** increased further after boost immunizations, suggesting the reinforcement of immune response against **1**. The globo H-specific IgG antibody titers (2783) of the day 21 antiserum of KLH conjugate **2** was about 17-fold lower than that of **1**. After four immunizations, the IgG antibody titers induced by **2** was only 29 383, *ca.* 2.4-fold lower than that of **1**. On the other hand, the titers of globo H-specific IgM antibodies induced by both conjugates were low (ESI†).

**Fig. 3:**
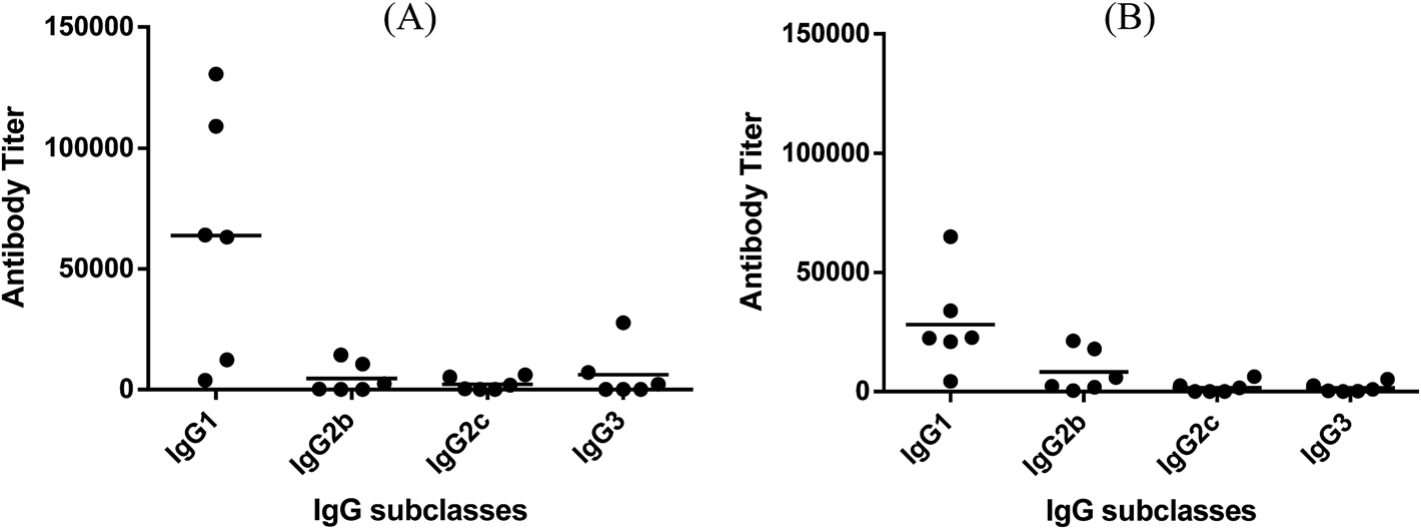
The titers of IgG antibody subclasses in individual antiserum collected from mice immunized with conjugates **1** (A) and **2** (B). Each dot represents the result of one mouse and the horizontal bar represents the average antibody titer for each group of six mice.

Fig. 3 depicts the ELISA results about various subclasses of anti-globo H IgG antibodies in the day 38 antiserum of each individual mouse inoculated with glycoconjugate **1** or **2**, as well as the group average. It was clear that conjugates **1** and **2** induced the similar patterns of immune responses, in both cases mainly IgG1 antibody (titers: 63 813 for **1** and 28 237 for **2**), as well as a lower level of IgG2b antibody (titers: 4578 for **1** and 8294 for **2**). Additionally, conjugate **1** also elicited some IgG3 antibody (titer: 6159), which is typical with MPLA conjugates.^26,28^

**Fig. 4:**
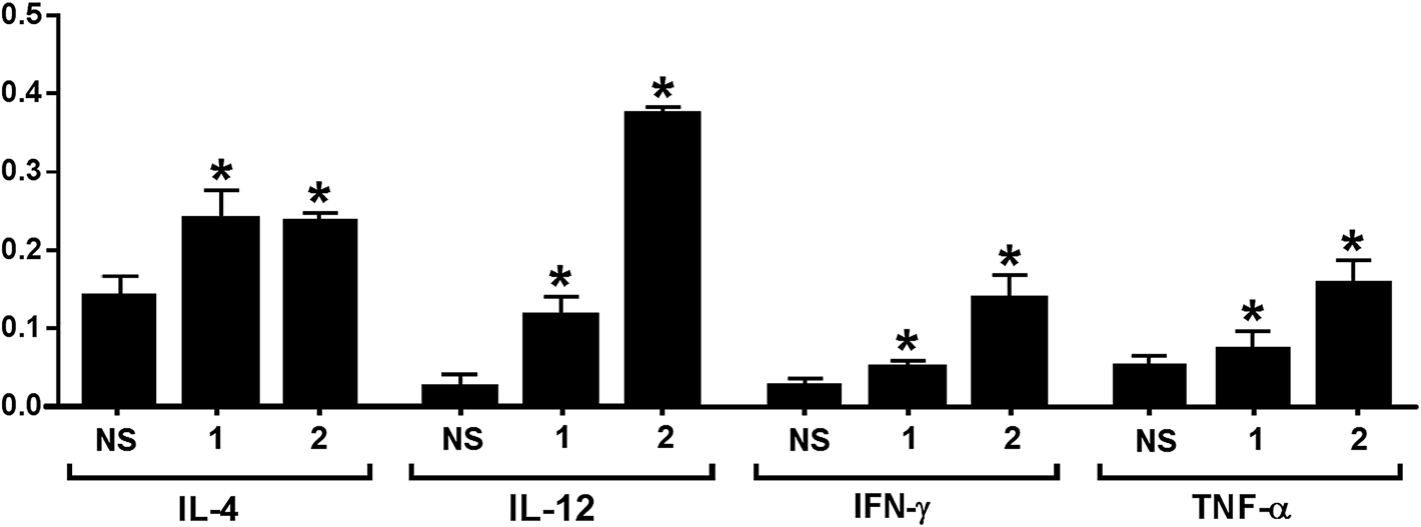
Relative intensities of IL-4, IL-12, IFN-γ, and TNF-α in the pooled normal mouse sera (NS) and the pooled day 38 antisera from mice immunized with conjugates **1** and **2**, respectively. The error bar represents the SD of two parallel experiments. *Compared to the result of NS, the difference is statistically significant.

The release of cytokines provoked by conjugates **1** and **2** was also analyzed. Compared to normal mouse sera, the expression levels of IL-3, IL-4, IL-9, IL-10, IL-12, IL-13, IFN-γ, MCP-1, MCP-5 and RANTES for **1** and that of GCSF, GM-CSF, IL-3, IL-4, IL-5, IL-6, IL-9, IL-10, IL-12, IL-13, IFN-γ, MCP-1, MCP-5, RANTES and TNF-α for **2** were elevated in the pooled antisera (ESI†). The relative expression levels of IL-4, IL-12, IFN-γ, and TNF-α elicited by conjugates **1** and **2** are depicted in Fig. 4. Generally, increased IL-4 expression indicates the activation of Th2 cell, which can help enhance B cell immune responses and antibody switch to IgG1.^57^ This result is consistent with that of ELISA (Fig. 3); therefore, both conjugates might have stimulated Th2 cell-mediated immunities. On the other hand, increased IL-12 expression suggests NK cell activation, and IFN-γ and TNF-α are produced by Th1 and/or CD8 cytotoxic T cells, which can activate macrophages and induce Ig antibody switch. The intensities of IL-12, IFN-γ, and TNF-α induced by **2** were higher than that induced by **1**. However, as cytokines are non-specific, it is difficult to tell whether the elevated cytokine expression induced by **2** was due to the protein carrier or due to the TACA moiety. Overall, the results of cytokine release analysis indicated that conjugates **1** and **2** induced T cell-mediated immunities.

The above results proved that the MPLA–globo H conjugate **1** could effectively elicit robust immune responses against globo H in mice in the absence of an external adjuvant and that it could elicit significantly faster and stronger immune responses than the corresponding KLH conjugate currently in clinical trial for cancer immunotherapy. The patterns of immune responses elicited by conjugates **1** and **2** were similar, namely that both elicited mainly IgG1 antibodies and some IgG2b antibodies, which is a good indication of T cell-mediated immunities.^58,59^ This conclusion was also supported by the results of cytokine analysis of the pooled antisera derived from mice immunized with conjugates **1** and **2**. Moreover, the IgG antibody titers increased with the number of boost immunizations for both glycoconjugates. The elicitation of strong IgG antibody responses and T cell-dependent immunities is critical for the therapeutic efficacy of cancer vaccines, since this is associated with antibody affinity maturation, improved antitumor activity, and long-term immunological memory. The significantly stronger T cell-dependent and IgG antibody immune responses induced by **1**, as compared to the KLH conjugate **2**, suggested the promise of **1** as a therapeutic cancer vaccine. It is also worth noting that the MPLA conjugate **1** elicited mainly IgG1 and IgG2b antibodies that are typically associated with protein conjugates. However, in previous studies, we observed that the MPLA conjugates of unnatural TACA analogs^26,28^ induced mainly carbohydrate-specific IgG3 antibodies.

### Antiserum binding to cancer cells

**Fig. 5:**
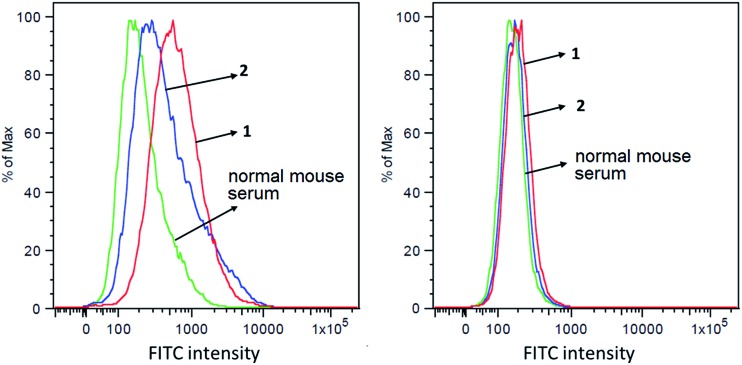
FACS assay results of the binding between MCF-7 (A) or SKMEL-28 (B) cancer cell and normal mouse serum (green), pooled antisera derived from mice immunized with conjugate **1** (red) or pooled antisera derived from mice immunized with conjugate **2** (blue), respectively.

The capabilities of antisera obtained with conjugates **1** and **2** to recognize and bind to target cancer cells were investigated by the fluorescence-activated cell sorting (FACS) technology. Breast cancer cell MCF-7, which expresses globo H,^60^ was used in this study, with melanoma cell SKMEL-28 that does not express globo H as a negative control. These two cell lines were individually cultured with normal mouse serum (the negative control) or antisera derived from mice immunized with **1** and **2**. Thereafter, cancer cells were incubated with fluorescein isothiocyanate (FITC)-labeled goat anti-mouse kappa antibody and were finally subjected to FACS analysis. As depicted in Fig. 5A, significant fluorescent peak shifts to the right were observed with MCF-7 cell treated with anti-**1** and anti-**2** sera as compared to the cell treated with normal mouse serum. In contrast, the fluorescent profiles of SKMEL-28 cell treated with normal mouse serum and with anti-**1** and anti-**2** sera did not exhibit a significant difference (Fig. 5B). These results demonstrated that the antibodies elicited by conjugates **1** and **2** could specifically target and bind to globo H-expressing cancer cells but not cells that do not express globo H. Furthermore, the median fluorescence intensity (MFI) of MCF-7 cells treated with anti-**1** serum (MFI: 580) was significantly higher than that of MCF-7 cells treated with anti-**2** serum (MFI: 367) (Fig. 5A), indicating increased binding events and/or affinity of antibodies in anti-**1** serum. This result was consistent with the ELISA results described above, namely that the antisera obtained with **1** had much higher antibody titers than the antisera obtained with **2**, and thereby had provided another piece of evidence supporting the conclusion that **1** could induce significantly stronger immunological responses in mice than **2**.

### Antibody-mediated complement-dependent cytotoxicity (CDC) to cancer cells

The anticancer activities mediated by antisera derived from mice inoculated with conjugates **1** and **2** were also evaluated with cancer cells MCF-7 and SKMEL-28. In this study, cancer cells were cultured with normal mouse serum or with the above-mentioned antisera in the presence of rabbit complements, and the induced cell lysis was then analyzed by the lactate dehydrogenase (LDH) assay (see Experimental section for details).

**Fig. 6:**
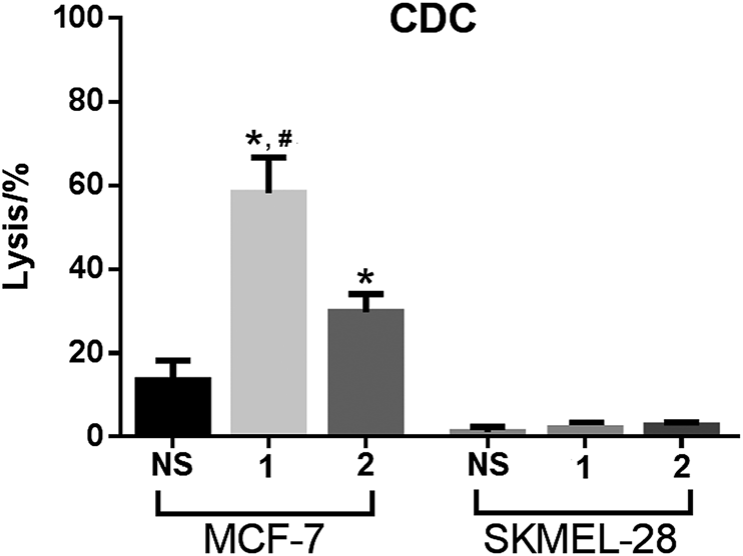
The results of antibody-mediated complement-dependent cytotoxicity to MCF-7 and SKMEL-28 cells, shown as cell lyses caused by treatment with complements and normal mouse serum (NS), pooled antisera derived from mice immunized with **1** or pooled antisera derived from mice immunized with **2**. The error bar shows the standard deviation of six parallel experiments. *Compared to the result of NS, the difference is statistically significant (*P* ≪ 0.01); ^#^compared to the result of anti-**2** sera, the difference is statistically significant (*P* ≪ 0.01).

As depicted in Fig. 6, under the non-optimized condition, the lysis rates of MCF-7 cell mediated by anti-**1** and anti-**2** sera were about 60% and 30%, respectively. In contrast, under the same condition, no antibody-mediated cytotoxicity to SKMEL-28 cell was observed. The results confirmed that the antisera raised by conjugates **1** and **2** mediated effective and specific CDC to cancer cells which express the globo H antigen. The results in Fig. 6 further demonstrated that anti-**1** sera mediated significantly stronger CDC to MCF-7 cell than anti-**2** sera under the same condition, supporting that **1** may be a better vaccine than **2** for cancer immunotherapy.

## Conclusion

Globo H is a hot topic in cancer immunological and immunotherapeutic studies, and globo H-based cancer vaccines have witnessed great progress in the past two decades.^9^ For example, the KLH–globo H conjugate as a therapeutic cancer vaccine for the treatment of breast, prostate, and other epithelial tumors has been on clinical trial.^14,20^ In spite of the promising development, these vaccines still have some serious issues, such as inconsistency in eliciting T cell-mediated immunity in cancer patients, mandatory usage of an adjuvant, emergence of immunotolerance to KLH, and difficulties in the control of conjugate quality. To address the issues, we have explored here a fully synthetic globo H-based conjugate vaccine using MPLA as a carrier molecule.

In addition to the conventional advantages of fully synthetic vaccines, such as well-defined structures, convenient characterization and easy quality control, the MPLA–globo H conjugate **1** had also exhibited some other useful properties as a therapeutic cancer vaccine. First, it elicited a faster and stronger immune response than the corresponding KLH conjugate **2**. A robust immune response against globo H was established in mice after immunization with **1** twice, while it took four times of immunization with **2** to develop a solid immune response, and under such condition the titers of induced globo H-specific antibodies were still significantly lower than that induced by **1**. A proposed explanation for this was that the strong immune response against KLH (the KLH-specific antibody titer was 293 919, *ca.* 12.7-fold higher than the globo H-specific antibody titer 23 177, ESI†) might have suppressed the immune response to the carbohydrate antigen.^16,17^ However, as a carrier molecule MPLA did not have this problem, since the MPLA-specific antibody titer (59 666) induced by the MPLA conjugate **1** was not significantly different from the globo H-specific antibody titer (63 038, ESI†). Second, the MPLA–globo H conjugate **1** was self-adjuvanting, thus it alone without the use of an external, traditional adjuvant was immunologically active. This would not only simplify its clinical use but also help stabilize its property and function and reduce side effects. It is worth noting that the liposomes of **1** formed with phosphatidylcholine (PC) and cholesterol were used in this research mainly for the purpose of increasing water solubility of **1** to obtain its homogeneous formulation. In the meantime, it was also reported that liposomes had some immunostimulative activities.^52,53^ However, a study showed that liposomes consisting of DSPC and cholesterol alone did not have immunostimulative activity, although negatively charged liposomes consisting of PC, cholesterol and dicetylphosphate (DCP) had immunostimulative activity.^61^ Clinical use of liposomes will not be likely to cause any problem, as liposomal drug delivery is now a mature and clinically widely adopted technology.^62^ Third, similar to the KLH conjugate **2**, **1** also elicited T cell-dependent immunity, which is very desirable for therapeutic cancer vaccines. Consequently, all of the immunological results have suggested that the globo H–MPLA conjugate **1** is a promising vaccine for cancer immunotherapy and it is worth additional investigation and development. This conclusion was further supported by the discovery that the antisera induced by **1** had significantly stronger binding to and CDC against globo H-expressing MCF-7 cancer cell than the antisera induced by the KLH conjugate **2** under the specified experimental conditions.

In addition, it is very interesting to notice that the MPLA conjugate **1** elicited mainly IgG1 and IgG2b antibodies instead of IgG3 antibodies previously observed with the MPLA conjugates of unnatural TACA analogs.^26,28^ IgG1 and IgG2b antibodies are usually induced by carbohydrate–protein conjugates, while IgG3 antibodies are often associated with non-peptide/protein conjugates of carbohydrates, such as glycolipids. The production of IgG1/IgG2 and IgG3 antibodies involves different immunological pathways.^59^ The reason for switching immunological pathways among different MPLA conjugates was not clear, but these results suggested that the immune response to a glycoconjugate was affected not only by the carrier molecule but also by the carbohydrate antigen involved, a discovery that is worthy further investigation.

## Experimental section

### Materials, reagents, and animals

CFA, DSPC, and rabbit complements were purchased from Sigma-Aldrich. MCF-7 and SKMEL-28 cancer cells, Dulbecco's Modified Eagle's Medium (DMEM) used for cell culture, and fetal bovine serum (FBS) were purchased from American Type Culture Collection (ATCC). Penicillin–streptomycin and trypsin–EDTA were purchased from Invitrogen. Alkaline phosphatase (AP)-linked goat anti-mouse kappa, IgM, IgG1, IgG2b, IgG2c, and IgG3 antibodies and FITC-labeled goat anti-mouse kappa antibody were purchased from Southern Biotechnology. Female C57BL/6J mice of 6–8 weeks old used for immunological studies were purchased from the Jackson Laboratory. LDH Cytotoxicity Detection Kit was purchased from Takara Bio Inc.

### General experimental methods

Chemicals and materials were obtained from commercial sources and were used as received without further purification unless otherwise noted. MS 4 Å was flame-dried under high vacuum and used immediately after cooling under a N_2_ atmosphere. Analytical TLC was carried out on silica gel 60 Å F_254_ plates with detection by a UV detector and/or by charring with 15% (v/v) H_2_SO_4_ in EtOH. NMR spectra were recorded on a 400, 500, or 600 MHz machine with chemical shifts reported in ppm (*δ*) downfield from tetramethylsilane (TMS) that was used as an internal reference.

### Compound **7**

To a stirred solution of **6** (12 mg, 5 μmol) and **5** (6 mg, 8 μmol) in DMF (1.5 mL) was added *N*-methylmorpholine (NMM, 6 μL, 54 μmol) at 0 °C. After the reaction mixture was stirred at rt overnight, DMF was removed in vacuum. The residue was purified on a TLC plate (MeOH/CH_2_Cl_2_/H_2_O/DMF 3 : 3 : 1 : 1, v/v) to get **7** as a white powder (8 mg, 55%). ^1^H NMR (600 MHz, CDCl_3_ : CD_3_OD : D_2_O = 3 : 3 : 1): *δ* 7.33–7.14 (m, 30H, ArH), 5.48 (t, *J* = 9.8 Hz, 1H, lipid-H-3′), 5.33–5.28 (m, 1H, lipid-H-3), 5.22–5.17 (m, 3H, 2H of lipid, and H-1′′′′′), 5.14–5.06 (m, 4H, (PhCH_2_O)_2_P), 1.98 (s, 3H, NHAc); 1.63–1.41 (m, 12H, lipid), 1.36–1.09 (br, 98H, 48 CH_2_, lipid), 0.96–0.77 (18H, 6 CH_3_, lipid). ^31^P NMR (400 MHz, CDCl_3_ : CD_3_OD : D_2_O = 3 : 3 : 1): *δ* –2.915; MS (ESI): calcd for C_176_H_276_N_5_O_54_P [M + 2Na]^+^ *m*/*z*, 1701.5; found, 1701.9.

### Compound **1**

A mixture of **7** (7.5 mg, 2.64 μmol) and 10% Pd–C (5.0 mg) in CH_2_Cl_2_ and MeOH (3 : 1, 4 mL) was stirred under an atmosphere of H_2_ at rt for 12 h. Thereafter, the catalyst was removed by filtration through a celite pad, and the celite pad was washed with a mixture of CH_2_Cl_2_, MeOH and H_2_O (1 : 1 : 1) and then with MeOH. The combined filtrates were concentrated in vacuum to afford glycoconjugate **1** as a white floppy solid (4.0 mg, 62%). ^1^H NMR (600 MHz, CDCl_3_ : CD_3_OD : D_2_O = 5 : 3 : 1): *δ* 5.13 (br, 1H, lipid-H-3′), 5.07–5.28 (br, 1H, lipid-H-3), 4.91 (br, 2H, 2H of lipid), 1.96 (s, 3H, NHAc); 1.81–1.56 (m, 12H, lipid), 1.53–1.11 (br, 98H, 48 × CH_2_, lipid), 1.05–0.85 (18H, 6 CH_3_, lipid). ^31^P NMR (400 MHz, CDCl_3_ : CD_3_OD : D_2_O = 5 : 3 : 1): *δ* –2.726; MS (ESI): calcd for C_134_H_245_KN_6_O_54_P [M + K + NH_4_]^2+^ *m*/*z*, 1436.3; found, 1436.9.

### Compound **8**

A mixture of hexasaccharide **5** (3 mg) and disuccinimidal glutarate (15 eq.) in DMF and 0.1 M PBS buffer (4 : 1, 0.5 mL) was stirred at rt for 6 h. The reaction mixture was concentrated under vacuum and the residue was washed with EtOAc 10 times. The resultant solid was dried under vacuum for 1 h to obtain activated oligosaccharide **8** that was directly used for conjugation with KLH and HSA, respectively. MALDI TOF MS (positive mode): calcd for C_49_H_79_N_3_O_35_ [M + Na]^+^ *m*/*z*, 1293.2; found, 1293.2.

### General procedure for conjugation of **8** with HSA and KLH

A mixture of the activated oligosaccharide **8** and KLH or HSA (5 mg) in 0.4 mL of 0.1 M PBS buffer was gently stirred at rt for 2.5 days. The mixture was purified on a Biogel A 0.5 column with 0.1 M PBS buffer as the eluent. The combined fractions containing the glycoconjugate indicated by the bicinchoninic acid (BCA) assay for proteins were dialyzed in distilled water for 1 day, and then lyophilized to give the desired glycoconjugates **2** and **3** as white floppy solids.

### Protocols to prepare vaccine formulations

Liposomal formulations of glycoconjugate **1** were prepared by a previously reported protocol.^26,28^ Briefly, after the mixture of conjugate **1** (0.5 mg, 0.17 μmol, for 30 doses), 1,2-distearoyl-*sn*-glycero-3-phosphocholine (DSPC) (0.87 mg, 1.1 μmol), and cholesterol (0.33 mg, 0.85 μmol) (in a molar ratio of 10 : 65 : 50) was dissolved in a mixture of CH_2_Cl_2_, MeOH and H_2_O (3 : 3 : 1, v/v, 2 mL), the solvents were removed under reduced pressure at 60 °C through rotary evaporation, which generated a thin lipid film on the vial wall. This film was hydrated by adding 3.0 mL of HEPES buffer (20 mM, pH 7.5) containing 150 mM of NaCl and shaking the mixture on a vortex mixer. The resultant suspension was sonicated with a sonicator for 20 min to afford the liposomal formulation used for immunizations. The average diameter of the liposomes was 1429.2 ± 249 (SD) nm with the polydispersity index (PDI) around 0.5832. The protocol for preparing CFA emulsions of the globo H–KLH conjugate **2** was similar to that reported in the literature.^56^ Generally, **2** (1.13 mg) was dissolved in 1.5 mL of 1 × PBS buffer and thoroughly mixed with CFA (1.5 mL) according to the manufacturer's instructions to generate the emulsion.

### Mouse immunization

Each group of six female C57BL/6J mice (6–8 weeks of age) was inoculated with subcutaneous (s.c.) injection of 0.1 mL of the liposomal formulation or the CFA emulsion of a specific conjugate on day 1. Following the initial inoculation, mice were boosted 3 times on day 14, day 21, and day 28 *via* s.c. injection of the same conjugate formulation. Mouse blood samples were collected prior to the initial immunization on day 0 and after immunization on day 21, day 27 and day 38, and were clotted to obtain sera that were stored at –80 °C before use. The animal protocol (#A 02-10-14) for this study was approved by the Institutional Animal Care and Use Committee (IACUC) of Wayne State University, and all of the animal experiments were performed in compliance with the relevant laws and institutional guidelines.

### ELISA protocol

ELISA plates were coated with a solution of the globo H–HSA conjugate **3** (2 μg mL^–1^, 100 μL) in the coating buffer (0.1 M bicarbonate, pH 9.6) at 37 °C for 1 h and then treated with a blocking buffer, *i.e.*, 1% BSA in PBS buffer containing 0.05% Tween-20 (PBST), followed by washing with PBST 3 times. Subsequently, a pooled or an individual mouse serum with serial half-log dilutions from 1 : 300 to 1 : 656 100 in PBS was added to the coated plates (100 μL per well). The plates were incubated at 37 °C for 2 h and then washed with PBST and incubated at rt for another hour with a 1 : 1000 diluted solution of AP-linked goat anti-mouse kappa, IgG1, IgG2b, IgG2c, IgG3, and IgM antibody (100 μL per well), respectively. Finally, the plates were washed with PBST and developed with a *p*-nitrophenylphosphate (PNPP) solution in buffer (1.67 mg mL^–1^, 100 μL) at rt for 1 h, followed by colorimetric readout using a microplate reader (ELX800, Bio-Tek instruments Inc.) at 405 nm wavelength. For titer analysis, the OD values were plotted against the serum dilution numbers to obtain a best-fit logarithm line. The equation of this line was used to calculate the dilution number at which an OD value of 0.1 was achieved, and this dilution number is defined as the antibody titer.

### Protocols for cytokine assay

Mouse cytokine antibody array-membrane (ab133993) was purchased from Abcam for detection of mouse cytokines in the day 38 antiserum according to the manufacturer's instruction. First, each membrane was blocked with the blocking buffer provided with the kit at room temperature for 30 min. Then, the membrane was incubated with the mouse serum (1 : 5 diluted in blocking buffer, 100 μL) at 4 °C overnight. After washing, the membrane was incubated with biotin-conjugated anti-cytokine antibodies at room temperature for 2 h. The membrane was washed again and incubated with HRP-conjugated streptavidin. The membrane was finally detected by using an X-ray film after addition of the chemiluminescence buffer. The signal intensity of positive controls was set as 1, and that of negative controls (background) as 0. The relative intensity of each cytokine in the normal mouse serum and each tested antiserum was calculated according to the equation shown below:

Relative intensity of a cytokine = (signal density of the cytokine spot – signal density of negative control)/(signal density of positive control – signal density of negative control).

### Protocols for FACS assay

Globo H-expressing MCF-7 and globo H-negative SKMEL-28 cell lines were used in the experiments. MCF-7 cell was incubated in ATCC-formulated Eagle's Minimum Essential Medium (EMEM) containing 10% FBS and 1% antibiotics, and SKMEL-28 cell was incubated in ATCC-formulated DMEM containing 10% FBS and 1% antibiotics. Both were harvested after treatment with trypsin–EDTA solution. Cells (about 1.0 × 10^6^) were washed twice with FACS buffer (PBS containing 5% FBS) and incubated with 50 μL of normal mouse serum (1 : 10 dilution) or a day 38 pooled antiserum (1 : 10 dilution) at 4 °C for 30 min. Thereafter, the cells were washed again with FACS buffer and incubated with FITC-linked goat anti-mouse kappa antibody (2 μL in 50 μL FACS buffer) at 4 °C for 30 min. Finally, cells were washed and suspended in 0.8 mL of FACS buffer for FACS analysis on a Becton Dickinson LSR II Analyzer at the Microscopy, Imaging and Cytometry Resources Core, Wayne State University.

### Protocols for CDC assay

CDCs were determined using the LDH Cytotoxicity Detection Kit according to manufacture's instructions. MCF-7 (1.0 × 10^4^ cells per well) and SKMEL-28 (1.5 × 10^4^ cells per well) cells were seeded in 96-well plates and then incubated at 37 °C overnight. After washing, 100 μL of a normal mouse serum (1 : 50 dilution) or a day 38 antiserum (1 : 50 dilution in medium) was added to each well, and the plates were incubated at 37 °C for 2 h. The cells were washed and then incubated with 100 μL of rabbit complement serum (1 : 10 dilution) at 37 °C for 1 h. For the low control (the background of LDH release), no mouse antiserum was added, while for the high control (maximum LDH release) the rabbit complement serum was replaced with 100 μL of 1% tritone-100. After incubation, 20 μL of supernatant was carefully transferred from each well into new 96-well plates containing 80 μL of PBS in each well. Then, 100 μL of the LDH Cytotoxicity Detection reagent was added to each well. The mixture was incubated at rt for 1 h. The optical absorption (*A*) of each well was read at 490 nm wavelength with a plate reader. The percentage of cell lysis is calculated according to the following the equation:Cell lysis% = (experimental *A* – low control *A*)/(high control *A* – low control *A*) × 100%where “experimental *A*” is the optical absorption at 490 nm of analyzed cells treated with a serum, “low control *A*” is the optical absorption of cells without serum treatment, and “high control *A*” is the absorption of cells completely lysed with a 1% triton solution.
