# Supplementary material for: A fully synthetic self-adjuvanting globo H-Based vaccine elicited strong T cell-mediated antitumor immunity
Source: Chem Sci. 2015 Sep 22;6(12):7112–21. doi: 10.1039/c5sc01402f (PMC4762603; doi:10.1039/c5sc01402f)
Supplement: Supplementary file 1 [file SC-006-C5SC01402F-s001.pdf]

Electronic Supplementary Material (ESI) for Chemical Science.
This journal is © The Royal Society of Chemistry 2015

Supporting Information

A Fully Synthetic and Self-Adjuvanting Globo H-Based Anticancer Vaccine Elicited Strong T Cell-Mediated Antitumor Immunity

Zhifang Zhou, Guochao Liao, Satadru S. Mandal, Sharad Suryawanshi, and Zhongwu Guo*
Department of Chemistry, Wayne State University, 1501 Cass Avenue, Detroit, Michigan 48202, United States
* Corresponding author: zwguo@chem.wayne.edu

Table of Contents:

I. Conjugate Synthesis and Analysis Experiments S1
II. Size Analysis of the MPLA Conjugate Liposomes S4
III. Calculated Antibody Titers of ELISA Experiments....S5
IV. Raw Data for the Cytokine Release Assays....S6
V. Assays of MPLA- and KLH Specific Antibodies S7
VI. NMR and MS Spectra of Synthesized Compounds....S9

I. Conjugate Synthesis and Analysis Experiments

General Experimental Methods. Chemicals and materials were obtained from commercial sources and were used as received without further purification unless otherwise noted. MS 4 Å was flame-dried under high vacuum and used immediately after cooling under a N_2 atmosphere. Analytical TLC was carried out on silica gel 60Å F_{254} plates with detection by a UV detector and/or by charring with 15% (v/v) H_2SO_4 in EtOH. NMR spectra were recorded on a 400, 500, or 600 MHz machine with chemical shifts reported in ppm (δ) downfield from tetramethylsilane (TMS) that was used as an internal reference.

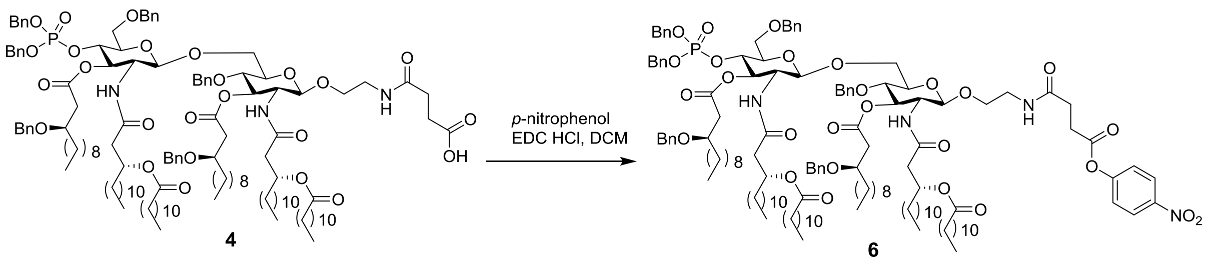

Compound 6. To a stirred solution of **4** (18 mg, 8 μmol) and *p*-nitrophenol (5.9 mg, 42 μmol) in CH_2Cl_2 (5 mL) was added EDC·HCl (8.2 mg, 42 μmol) in an ice bath. After the mixture was stirred at rt for 5 h, it was diluted with CH_2Cl_2, washed with brine, dried over anhydrous Na_2SO_4, and condensed in vacuum. The residue was purified on a TLC plate (MeOH/CH_2Cl_2, 1:20, v/v) to give the activated ester **6** as a white floppy solid (16 mg, 83.5%). Its spectroscopic data were identical to that reported in the literature (Z. Zhou, M. Mondal, G. Liao and Z. Guo, *Org. Biomol. Chem.*, 2014, **12**, 3238-3245).

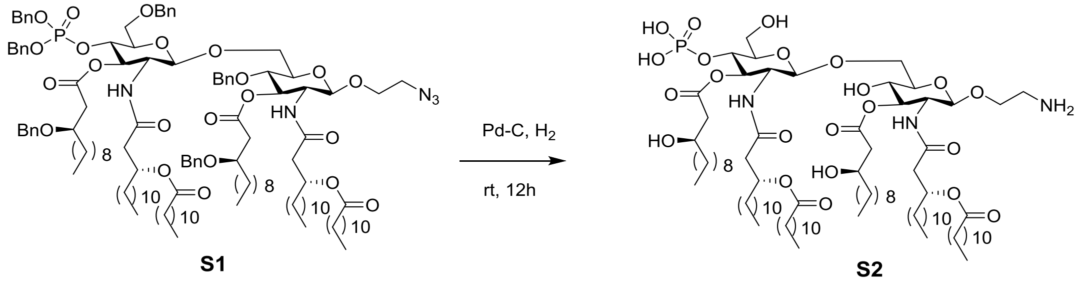

Compound S2. A mixture of **S1** (10.0 mg, 4.46 μmol, Z. Zhou, M. Mondal, G. Liao and Z. Guo, *Org. Biomol. Chem.*, 2014, **12**, 3238-3245) and 10% Pd-C (10.0 mg) in CH_2Cl_2-MeOH (3:1, 8 mL) was stirred under an atmosphere of H_2 at rt for 12 h. Thereafter, the catalyst was removed by filtration through a Celite pad, and the Celite pad was subsequently washed with CH_2Cl_2-MeOH-H_2O (3:3:1). The combined filtrates were concentrated in vacuum, to give **S2** as a yellow solid (7.0 mg, 93.7%). ^{1}H NMR (600 MHz, $CDCl_3$:CD_3OD:D_2O=3:3:1): δ 5.05-4.90 (br, 2H), 4.88-4.80 (br, 1H), 4.46-4.37 (br, 1H), 3.85-3.80 (br, 4H), 3.80-3.75 (m, 3H), 3.75-3.60 (br, 1H), 3.60-3.42 (m, 2H), 3.41-3.20 (m, 3H), 2.98 (br, 1H, ½$\underline{CH_2}$$NH_2$), 2.90 (br, 1H, ½$\underline{CH_2}$$NH_2$), 2.43-2.00 (m, 12 H, lipid), 1.50-0.95(br, 108 H, 54 x CH_2, lipid), 0.81-0.60 (18 H, 6 x CH3, lipid). ^{13}C NMR (150 MHz, $CDCl_3$:CD_3OD:D_2O=3:3:1): δ 173.71, 173.57, 172.34, 171.32, 171.00, 101.53, 75.18, 74.02, 71.26, 70.87, 68.60, 68.15, 60.03, 53.68, 52.87, 48.96, 48.81, 48.67, 48.52, 48.24, 48.10, 42.01, 41.60, 41.13, 40.98, 40.33, 37.27, 36.94, 34.27, 33.90, 33.80, 31.68, 31.14, 31.05,

29.93, 29.87, 29.82, 29.42, 29.32, 29.29, 29.25, 29.12, 28.98, 28.95, 25.34, 25.25, 25.05, 25.00, 24.86, 24.81, 22.42, 13.71; ^{31}P NMR (400 MHz, $CDCl_3$:CD_3OD:D_2O=3:3:1): δ 0.405; HRMS (ESI, TOF): calcd. For $C_{90}H_{171}N_3O_{22}P$ $[M+H]^+$ m/z, 1677.2091; found, 1677.2091. (**S2** was used as the capture reagent for ELISA analysis of MPLA-specific antibodies.)

Analysis of the carbohydrate loading of glycoconjugates (R. E. Wrolstad, T. E. Acree and E. A. Decker, *Current Protocols in Food Analytical Chemistry*, John Wiley & Sons, Inc., 2001): The sugar calibration curve was prepared using a standard solution of fucose, galactose, *N*-acetylgalactosamine, and glucose (1.2 mg/mL in 1/3/1/1 molar ratio) in distilled water. Aliquots were transferred to 10 dry 10 ml tubes in 5 µL increments ranging from 5 to 50 µL. In another 10 mL test tube, accurately weighed samples of the glycoconjugates to be analyzed were placed. At this point, all the tubes should contain between 5 to 50 µg of sugar, and one should contain an unknown amount of sugar to be determined. To all of the tubes were sequentially added 500 µL of 4% phenol and 2.5 mL of 96% sulfuric acid. The glycosyl linkages were cleaved and a colored complex was formed in this step. Solutions were transferred from the test tubes to cuvettes and measured at the wavelength of 490 nm. The calibration curve was obtained by plotting *A*490 against the weight (µg) of sugar in the standard samples. The amount of sugar present in each unknown sample was calculated based on the *A*490 of the unknown sample against the calibration curve, while the free proteins KLH and HSA were used as blank controls for conjugates **2** and **3**, respectively. The carbohydrate loading of each glycoconjugate was calculated according to the following equation, and the results for KLH conjugate **2** and HSA conjugate **3** were 8.0% and 14.0%, respectively.

Carbohydrate loading % = sugar weight in a tested sample/total weight of the sample × 100%

Procedure for SDS-PAGE analysis of glycoconjugates: The mixture of a glycoconjugate (1 µg) in 0.1 M PSB buffer (1 µl), LDS NuPAGE® sample buffer (4 ×, 2.5 µl), reducing agent (10×, 1 µl), and deionized water (5.5 µl) was heated at 70 °C for 10 min, and loaded onto the sodium dodecyl sulfate polyacrylamide gel. The upper buffer chamber was filled with 200 ml of 1 × SDS running buffer containing 500 µl of NuPAGE® antioxidant, and the lower buffer chamber was filled with 600 ml of 1 × SDS running buffer. The gel was run at 200 V for 2 h, and stained with Coomassie brilliant blue R-250 for 8 h (Ref: NuPAGE® technical instructions of Invitrogen).

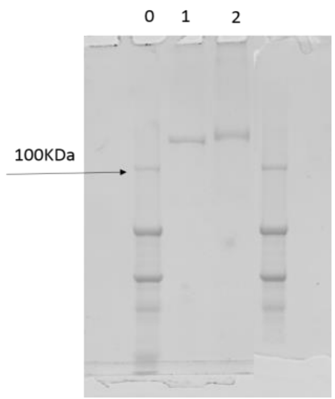

Figure S1. The SDS-PAGE results. Lane 0: molecular markers; Lane 1: KLH protein; Lane 2: KLH-Globo H conjugate **2**.

II. Size Analysis of the MPLA Conjugate Liposomes

The liposomal formulation of Globo H-MPLA conjugate **1** prepared for the immunization of mice was subjected dynamic light scattering (DLS). The sample was tested five times, and the results are listed in Table S1. It was concluded that the average diameter of the liposome was 1,429.2 ± 249 (standard deviation) nm with the polydispersity index (PDI) around 0.5832 (Figure S2).

Table S1. DLS analysis results for the liposomal formulation of Globo H-MPLA conjugate **1**

test	PDI	Size (d, nm)	% volume	Width (d, nm)
# 1	0.54	1503	96.5	225.2
# 2	0.472	1816	92.1	402.7
# 3	0.704	1366	100	173.2
# 4	0.521	1302	96	203.1
# 5	0.679	1159	94.1	173.7
**Ave**	**0.5832**	**1429.2**	**95.74**	
SD	0.1023	249.12	2.947	

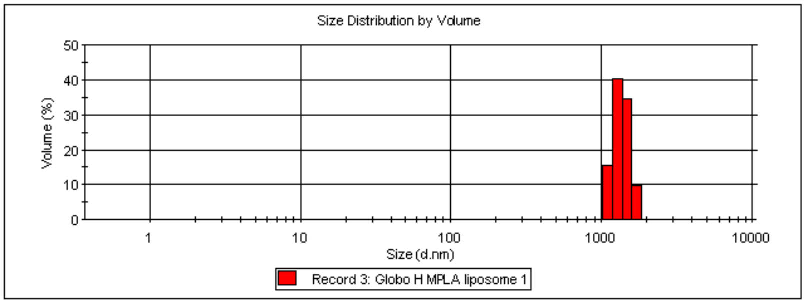

Figure S2. Size distribution of the liposomes of conjugate **1** based on DLS volume analysis

III. Calculated Antibody Titers of ELISA Experiments

Table S2. The total antibody titers of pooled antisera induced by conjugates **1** and **2** (Figure 2A)

	**1**			**2**		
	Mean	SD	N	Mean	SD	N
d0	0	0	3	0	0	3
d21	47824	3211	3	3299	481	3
d27	57648	2452	3	10937	2417	3
d38	63038	1595	3	29128	3110	3

Table S3. The IgG antibody titers of pooled antisera induced by conjugates **1** and **2** (Figure 2B)

	**1**			**2**		
	Mean	SD	N	Mean	SD	N
d0	0	0	3	0	0	3
d21	46449	7768	3	2783	655	3
d27	65577	850	3	8273	2600	3
d38	69406	1584	3	29383	3326	3

Table S4. The antibody titers of IgG subclasses in individual antiserum induced by **1** (Figure 3A)

mouse	1	2	3	4	5	6	Mean
IgG1	108952	3853	63146	12321	64011	130594	63813
IgG2b	10552	1	155	12	2430	14317	4578
IgG2c	6039	0	291	1751	55	5232	2228
IgG3	2177	0	2	14	7092	27667	6159

Table S4. The antibody titers of IgG subclasses in individual antiserum induced by **2** (Figure 3B)

mouse	1	2	3	4	5	6	Mean
IgG1	20952	22450	4333	22678	33973	65033	28237
IgG2b	21324	1772	2269	5963	17979	454	8294
IgG2c	22	2601	6299	15	1536	1	1746
IgG3	210	0	253	5148	2623	911	1524

Table S5. The antibody titers of IgM in individual antiserum induced by conjugate **1** and **2**

mouse	1	2	3	4	5	6	Mean
Conjugate **1**	2932	401	0	4866	39471	101153	24804
Conjugate **2**	28681	20618	16195	8	2	19789	14216

IV. Raw Data for the Cytokine Release Assays

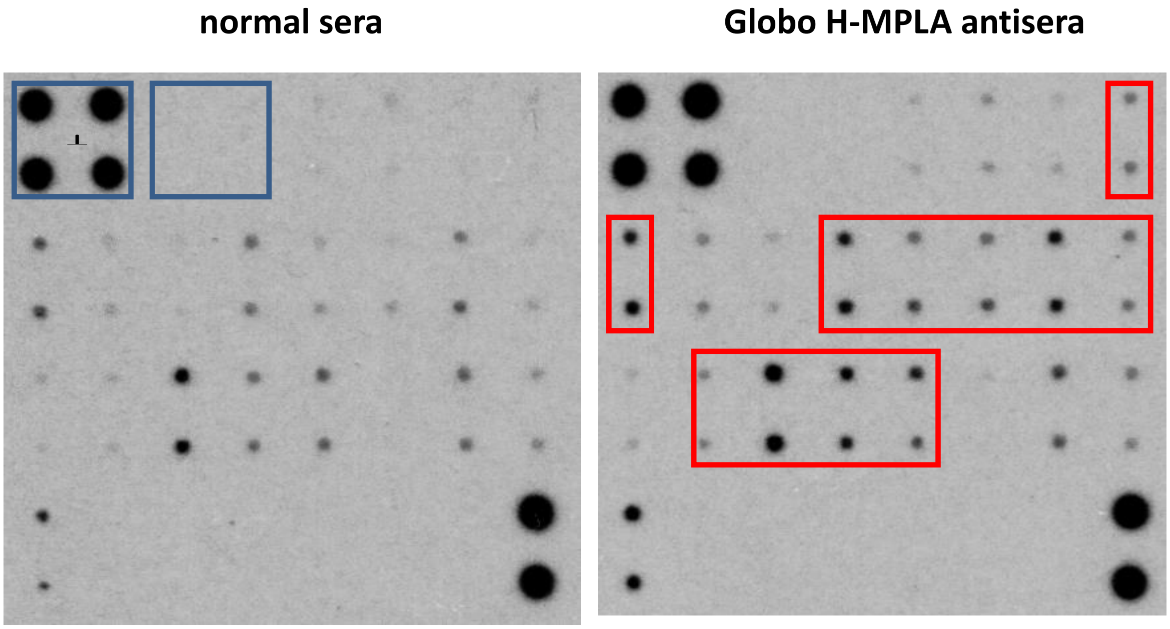

Figure S3. The expression levels of cytokines, including IL-3, IL-4, IL-9, IL-10, IL-12, IL-12p70, IL-13, IFN-γ, MCP-1, MCP-5, and RANTES, have significantly increased in the antisera derived from mice immunized with Globo H-MPLA **1**, compared to that of normal mouse sera.

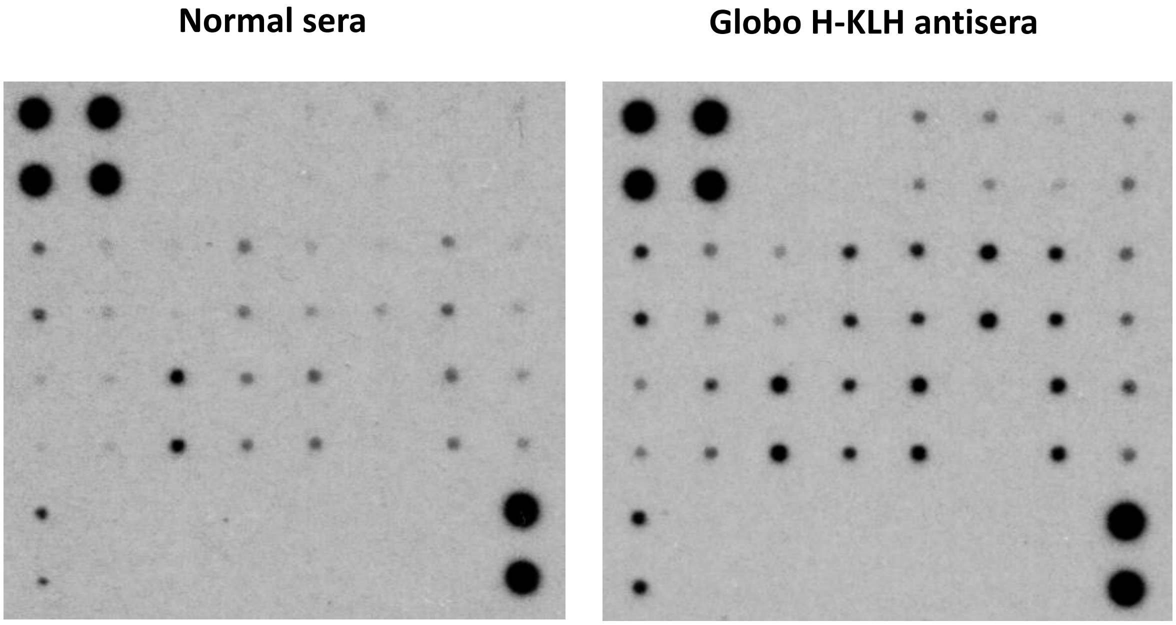

Figure S4. The expression levels of cytokines, including GCSF, GM-CSF, IL-3, IL-4, IL-5, IL-6, IL-9, IL-10, IL-12, IL-12p70, IL-13, IFN-γ, MCP-1, MCP-5, RANTES, and TNF-α, have significantly increased in the antisera derived from mice immunized with Globo H-MPLA **1**, compared to that of normal mouse sera.

	A	B	C	D	E	F	G	H
1	Pos	Pos	Neg	Neg	GCSF	GM-CSF	IL-2	IL-3
2	Pos	Pos	Neg	Neg	GCSF	GM-CSF	IL-2	IL-3
3	IL-4	IL-5	IL-6	IL-9	IL-10	IL-12 p40p70	IL-12p70	IL-13
4	IL-4	IL-5	IL-6	IL-9	IL-10	IL-12 p40p70	IL-12p70	IL-13
5	IL-17	IFN-γ	MCP-1	MCP-5	RANTES	SCF	sTNFRI	TNF-α
6	IL-17	IFN-γ	MCP-1	MCP-5	RANTES	SCF	sTNFRI	TNF-α
7	Thrombopoietin	VEGF	BLANK	BLANK	BLANK	BLANK	BLANK	Pos
8	Thrombopoietin	VEGF	BLANK	BLANK	BLANK	BLANK	BLANK	Pos

Figure S5. The membrane map for experiments to obtain the results in Figures S3 and S4.

V. Assays of MPLA- and KLH Specific Antibodies

MPLA-specific antibody titer was determined by ELISA, similar to that used to measure other antibodies but using MPLA as the capture reagent to coat the NUNC PolySorp™ 96-well plates. The MPLA derivative **S2** used for coating plates was dissolved in 0.2% triethylamine to get a final concentration of 0.03 mg/ml. After the solvent was evaporated, the plates were treated with blocking buffer following the normal protocol of ELISA. The MPLA-specific total antibody titer of the day 38 serum pooled from mice immunized with Globo H-MPLA conjugate **1** was 59,666, as compared to the Globo H-specific total antibody titer of 63,038 (Figure S6).

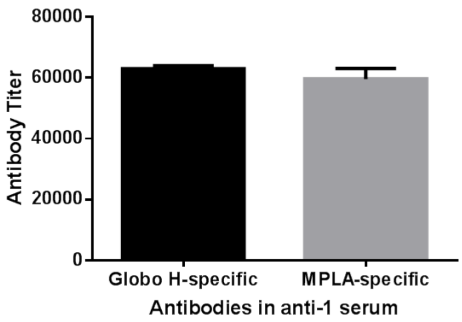

Figure S6. Titers of the Globo H- and MPLA-specific total antibodies in the pooled antiserum induced by conjugate **1**. Data are presented as mean ± SEM. The difference of Globo H- and MPLA-specific total antibody titers is not statistically significant.

KLH-specific antibody titer was determined by ELISA, similar to that used to measure other antibodies but using KLH as the capture reagent to coat the plates. KLH powder was dissolved in the coating buffer (0.1 M bicarbonate, pH 9.6) to get a final concentration of 2 μg/mL. Each well of the plates was treated with 100 μL of KLH coating solution at 37 °C for 1 h and then with a blocking buffer following the normal protocol of ELISA. The KLH-specific antibody titer of the day 38 antiserum pooled from mice immunized with Globo H-KLH conjugate **2** was 293,919, and its Globo H-specific antibody titer was 23,177 (Figure S7). Evidently, the KLH conjugate provoked much stronger anti-KLH antibody response than the anti-globo H antibody response.

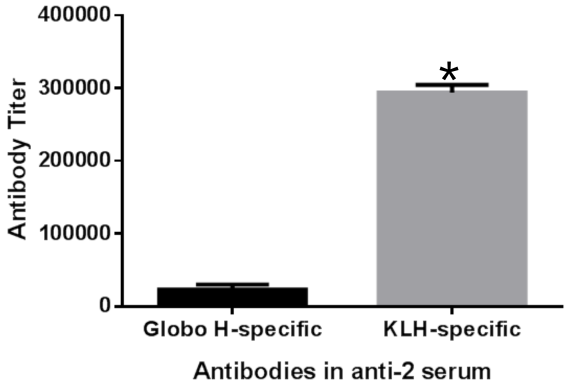

Figure S7. Titers of the Globo H- and KLH-specific antibodies in the pooled antiserum induced by conjugate **2**. Data are presented as mean ± SEM. * Compared to the Globo H-specific total antibody titer, the difference is statistically very significant ($P < 0.0001$).

VI. NMR and MS Spectra of Synthesized Compounds

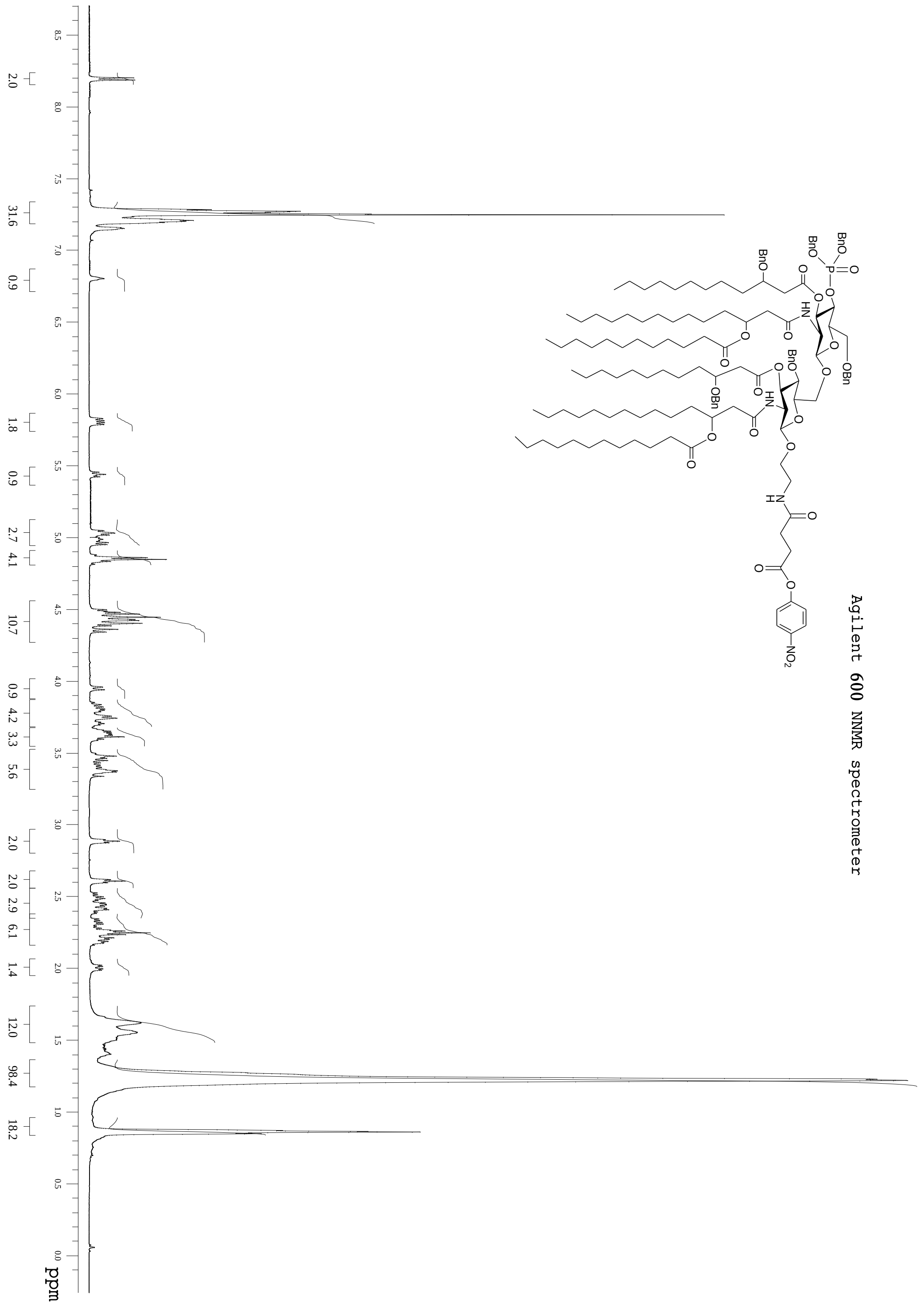

^{1}H NMR Spectrum of compound **6** ($CDCl_3$, 600 MHz)

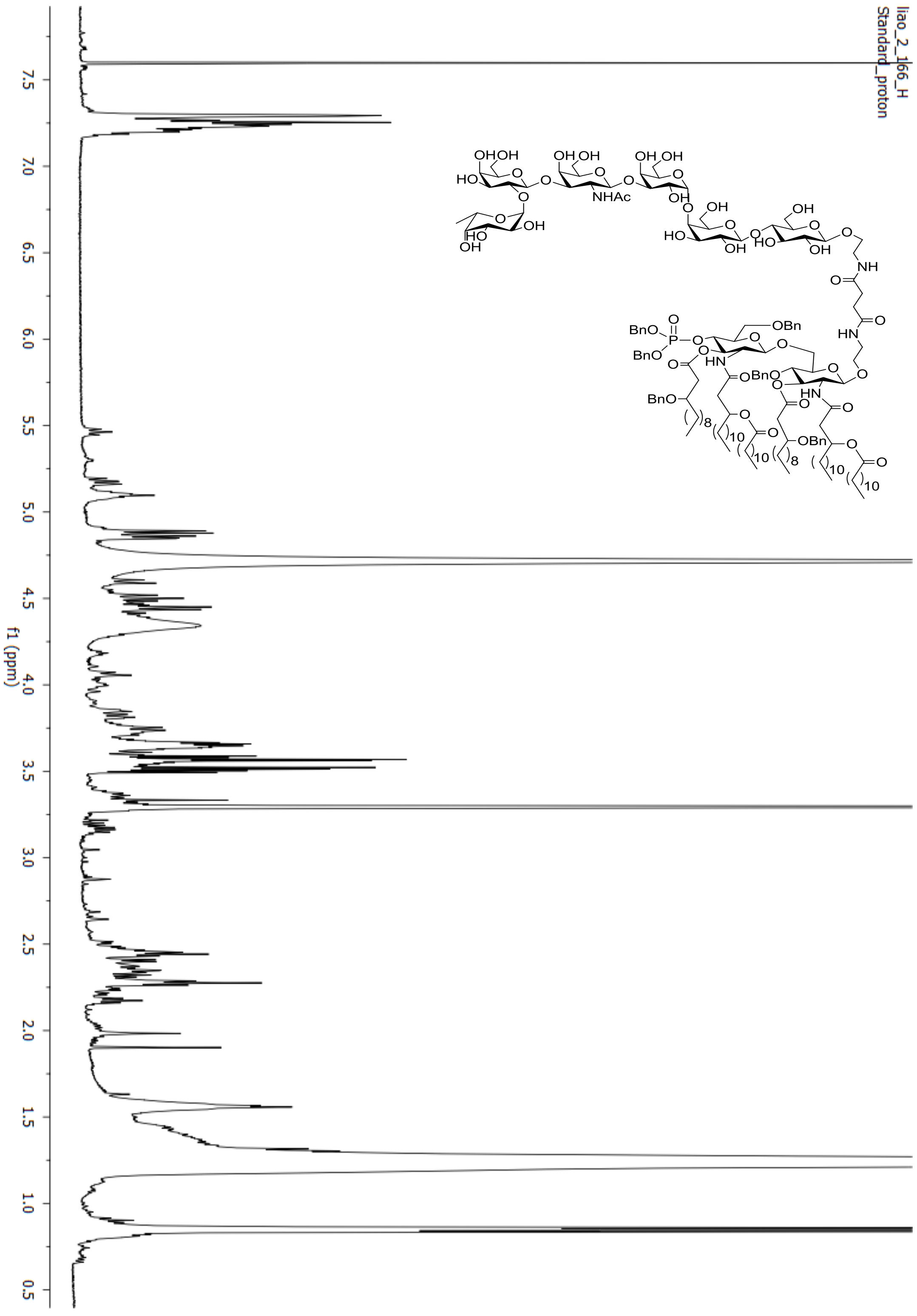

^{1}H Spectrum of compound **7** [$CDCl_3$:CD_3OD:D_2O (3:3:1) 600 MHz]

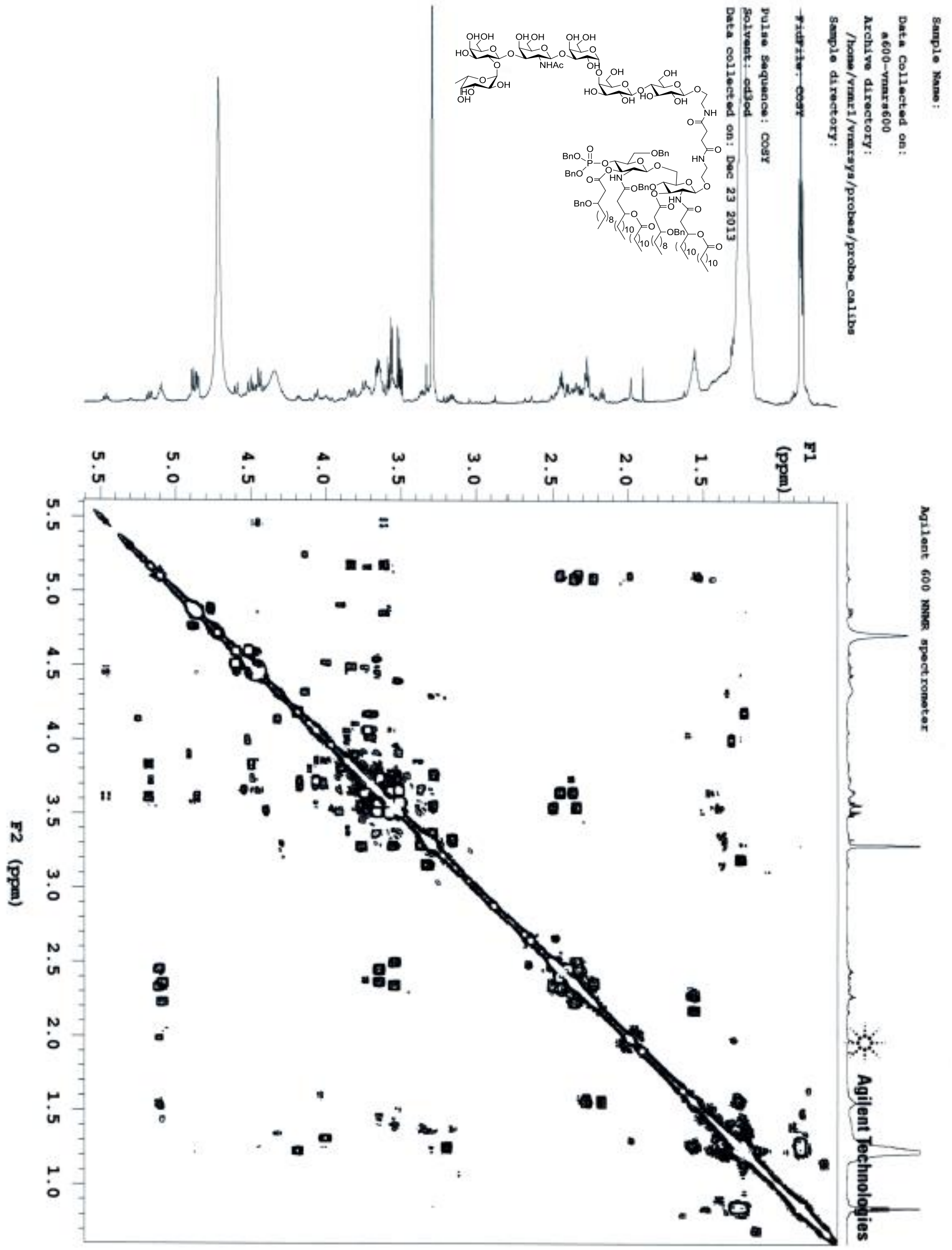

^1H-^1H COSY Spectrum of compound **7** [$CDCl_3$:CD_3OD:D_2O (3:3:1) 600 MHz]

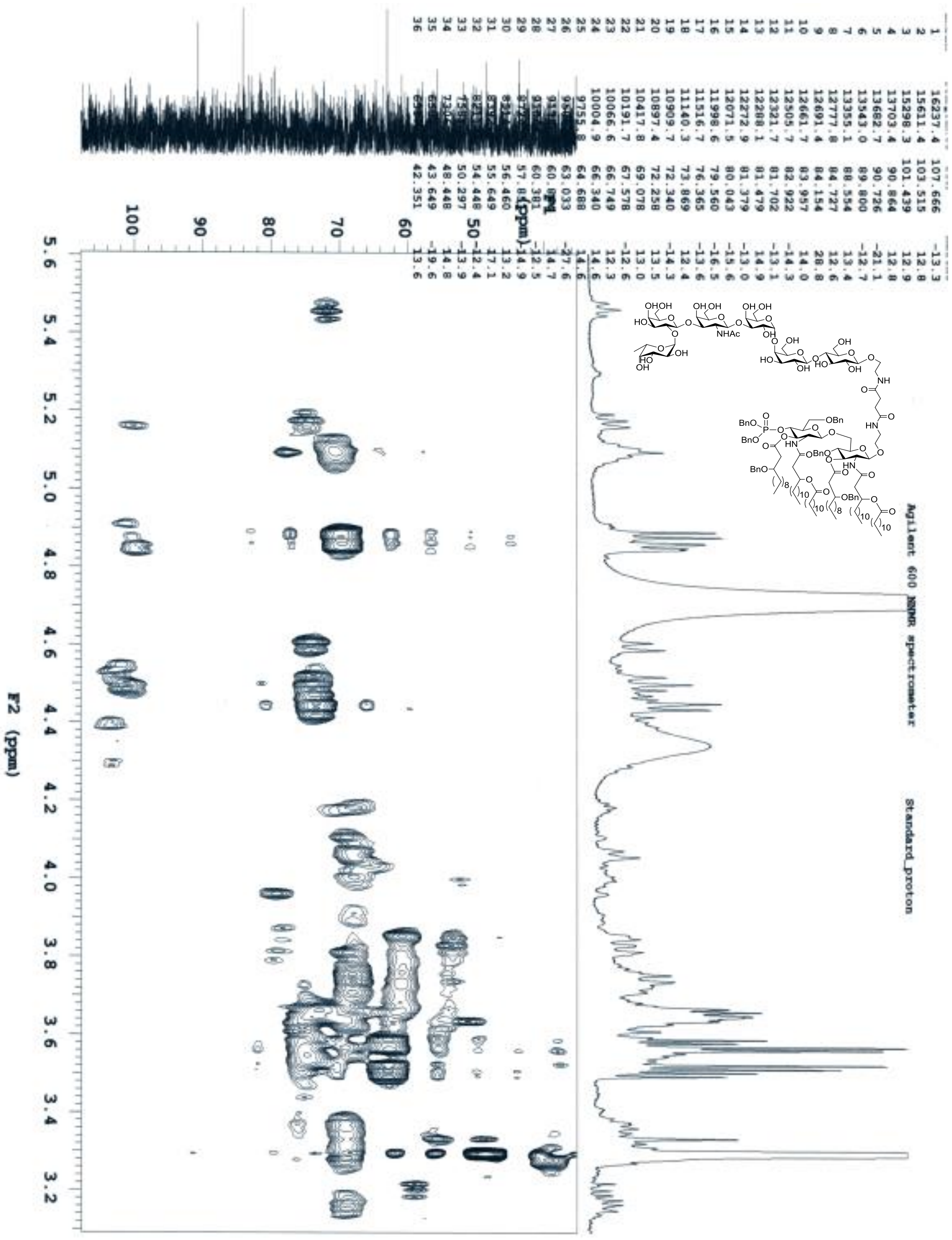

^{1}H-^{13}C HMQC NMR Spectrum of compound **7** [$CDCl_3$:CD_3OD:D_2O (3:3:1) 600/150 MHz]

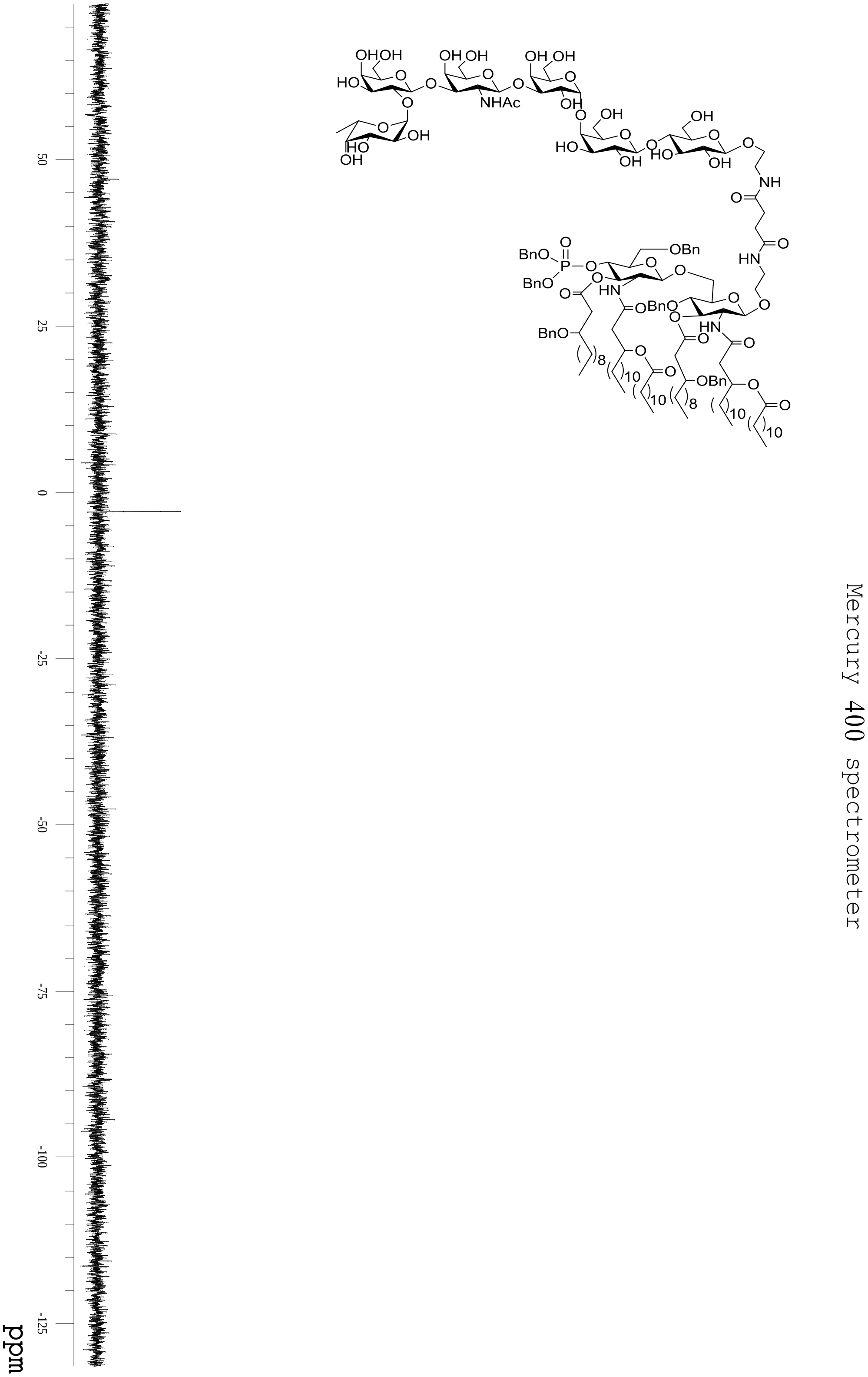

^{31}P NMR Spectrum of compound **7** [$CDCl_3$:CD_3OD:D_2O (3:3:1) 400 MHz]

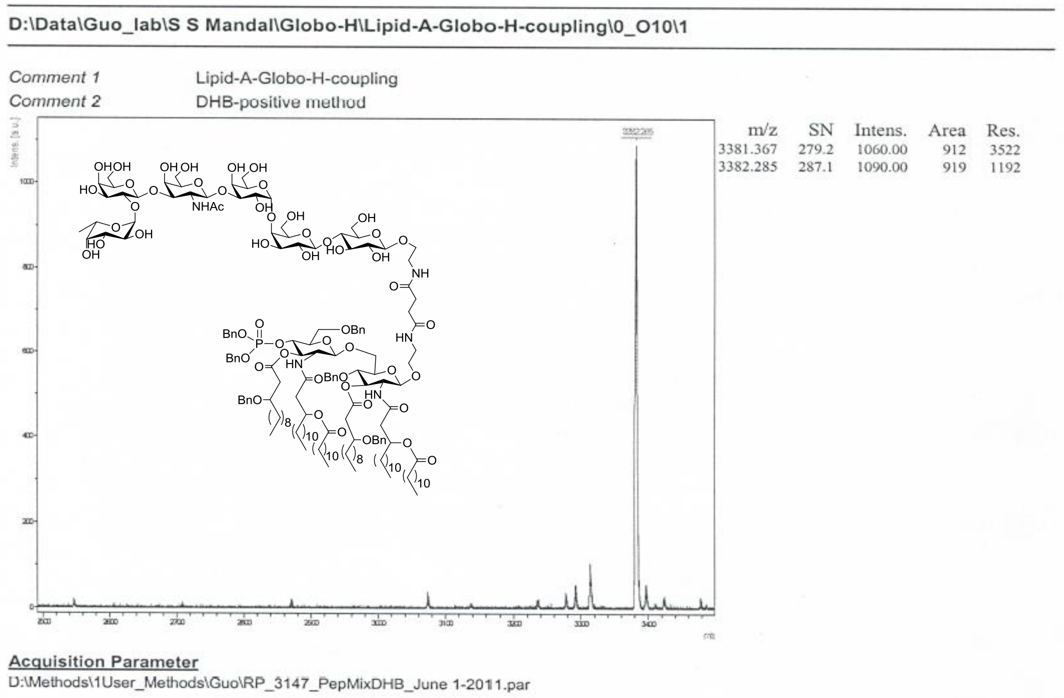

MALTI-TOF MS spectrum of compound **7**

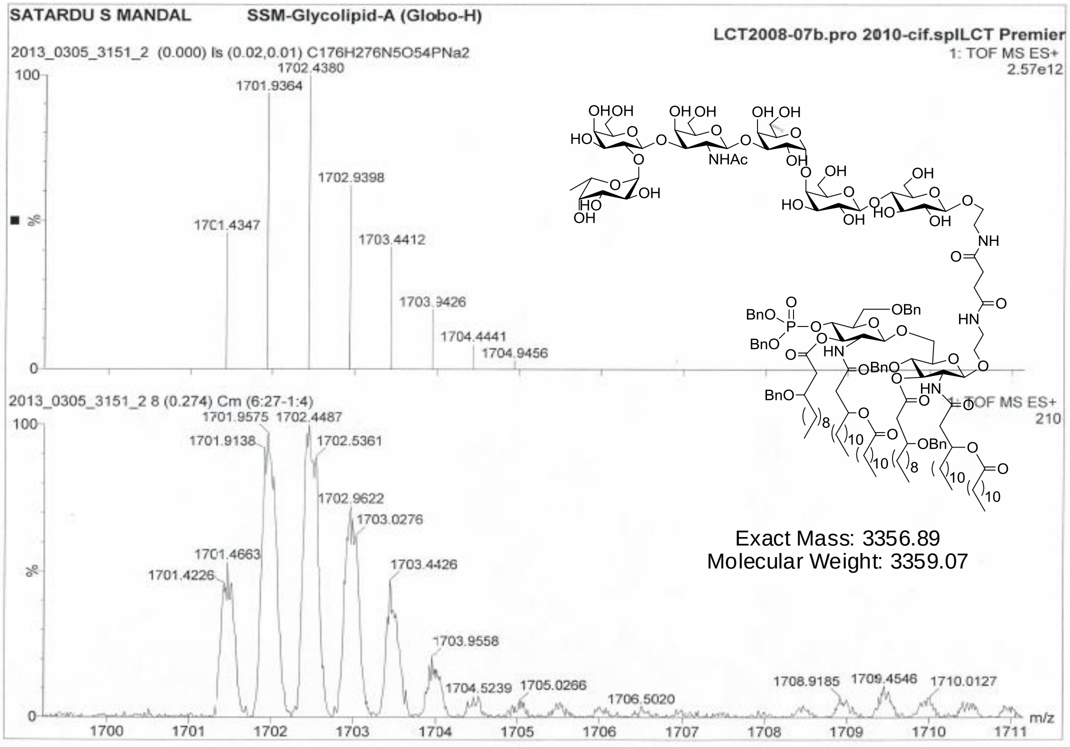

HRMS (ESI MS) spectrum of compound **7**

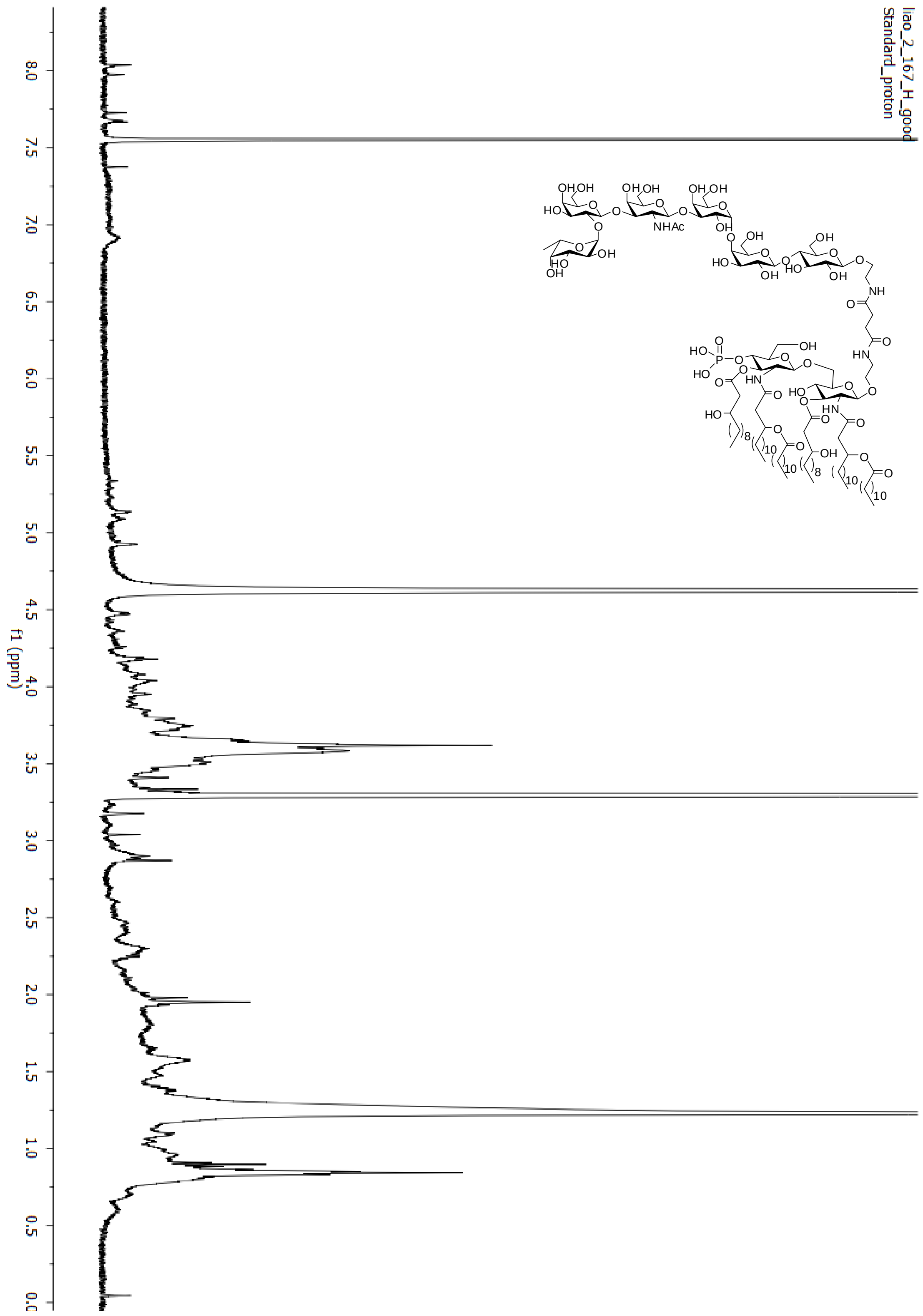

^{1}H NMR Spectrum of compound **1** [$CDCl_3$:CD_3OD:D_2O (5:3:1) 600 MHz]

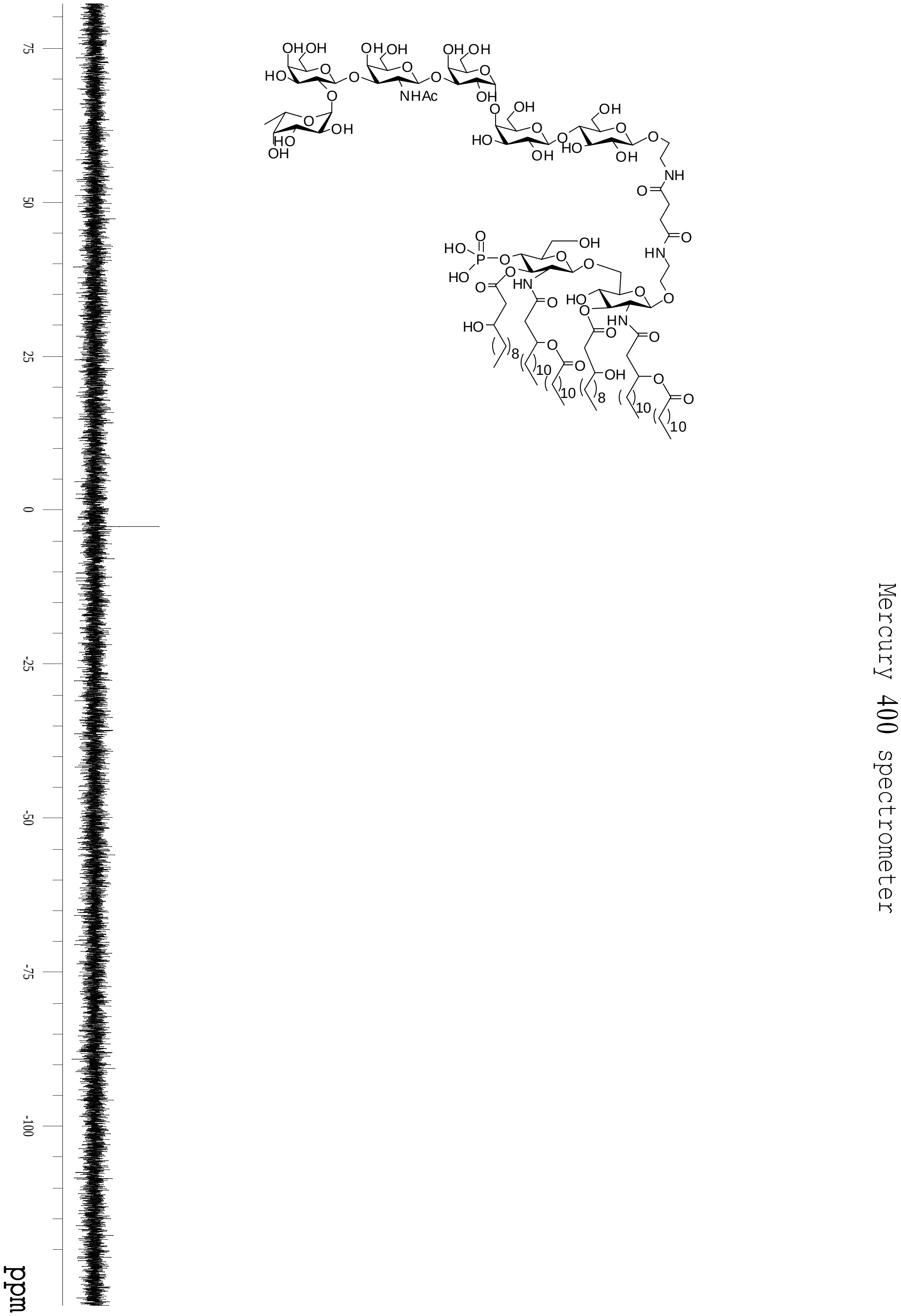

^{31}P NMR Spectrum of compound **1** [$CDCl_3$:CD_3OD:D_2O (5:3:1) 400 MHz]

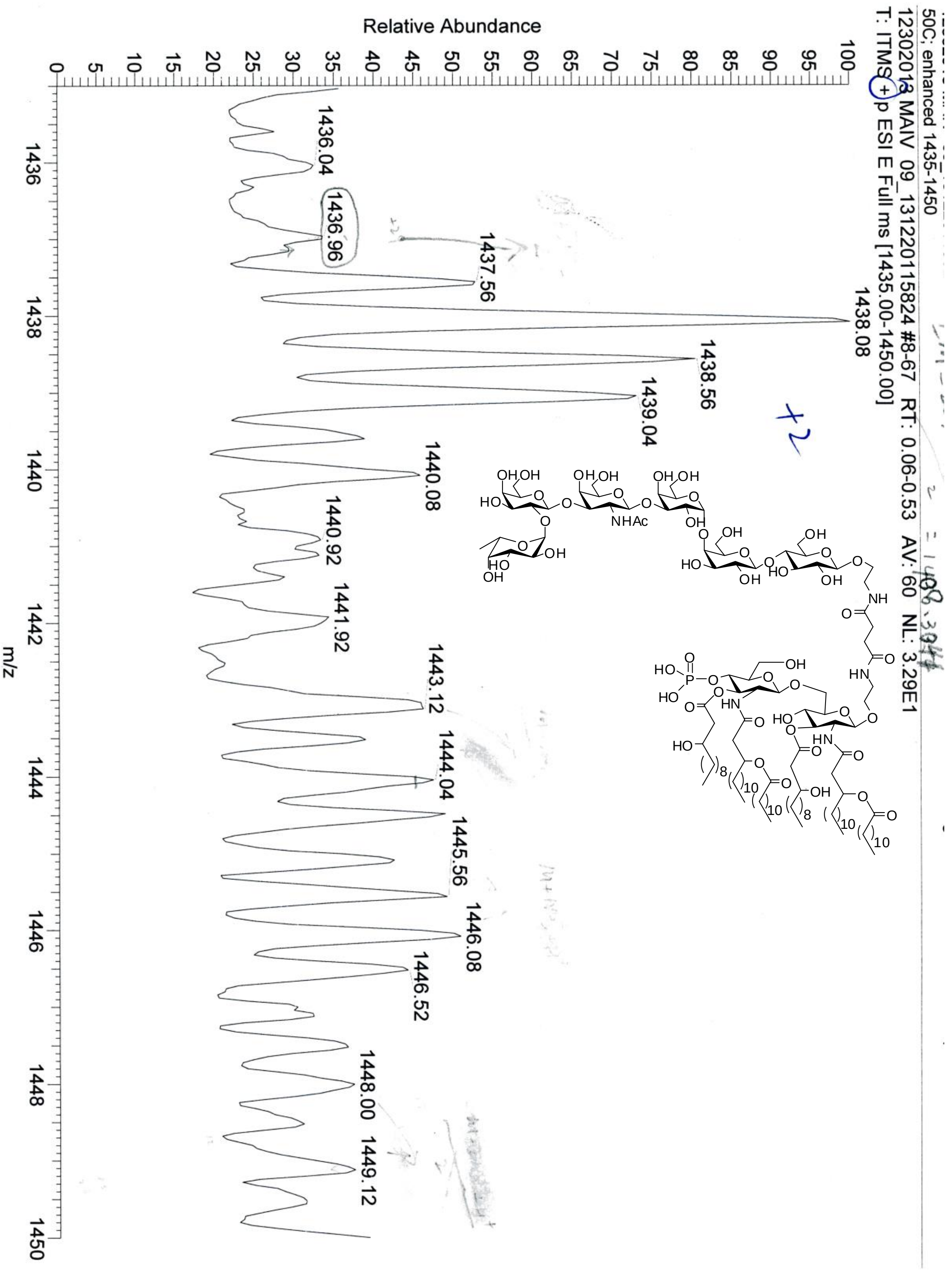

MS (ESI MS) spectrum of compound **1**

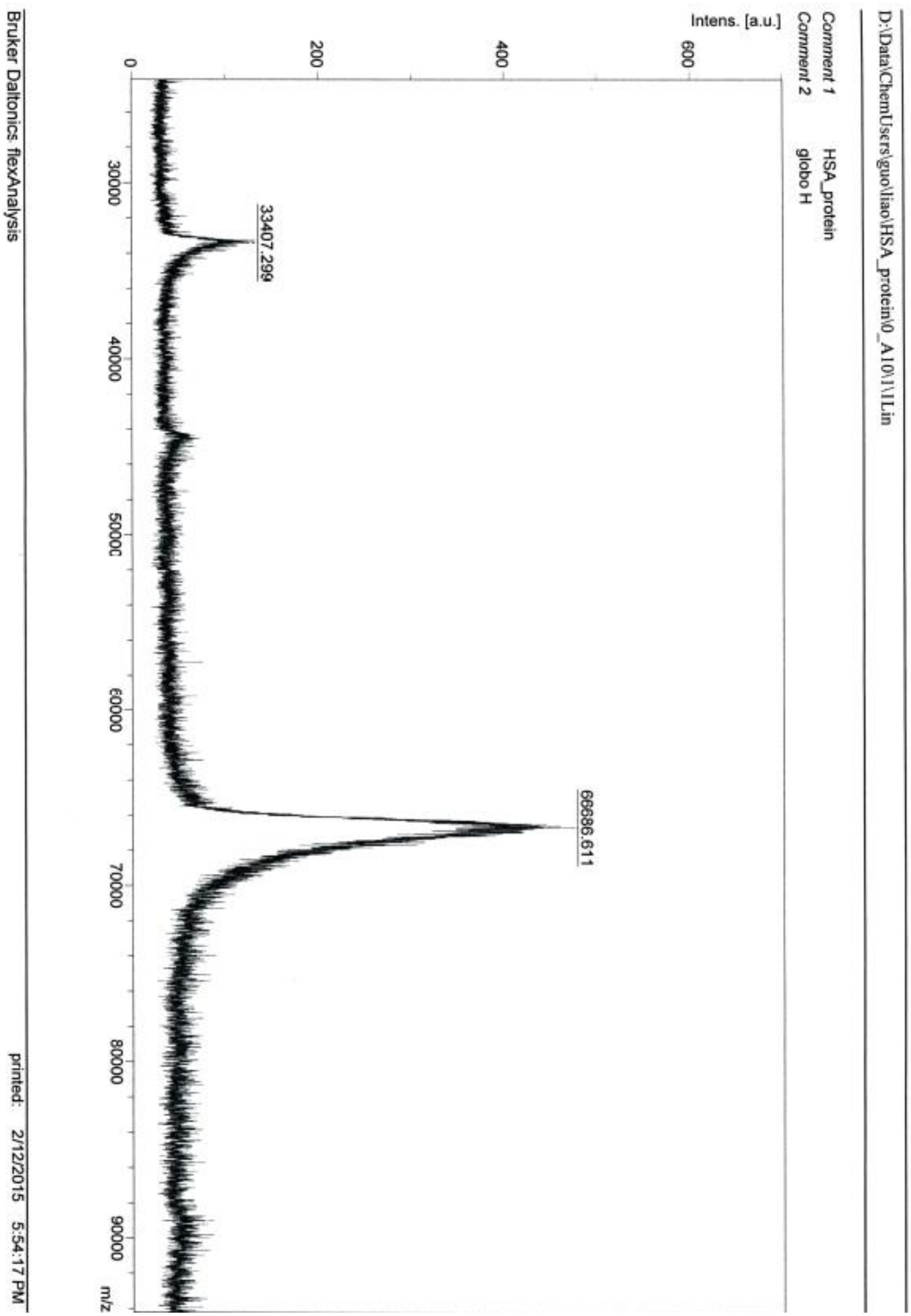

MALTI-TOF MS spectrum of HSA protein

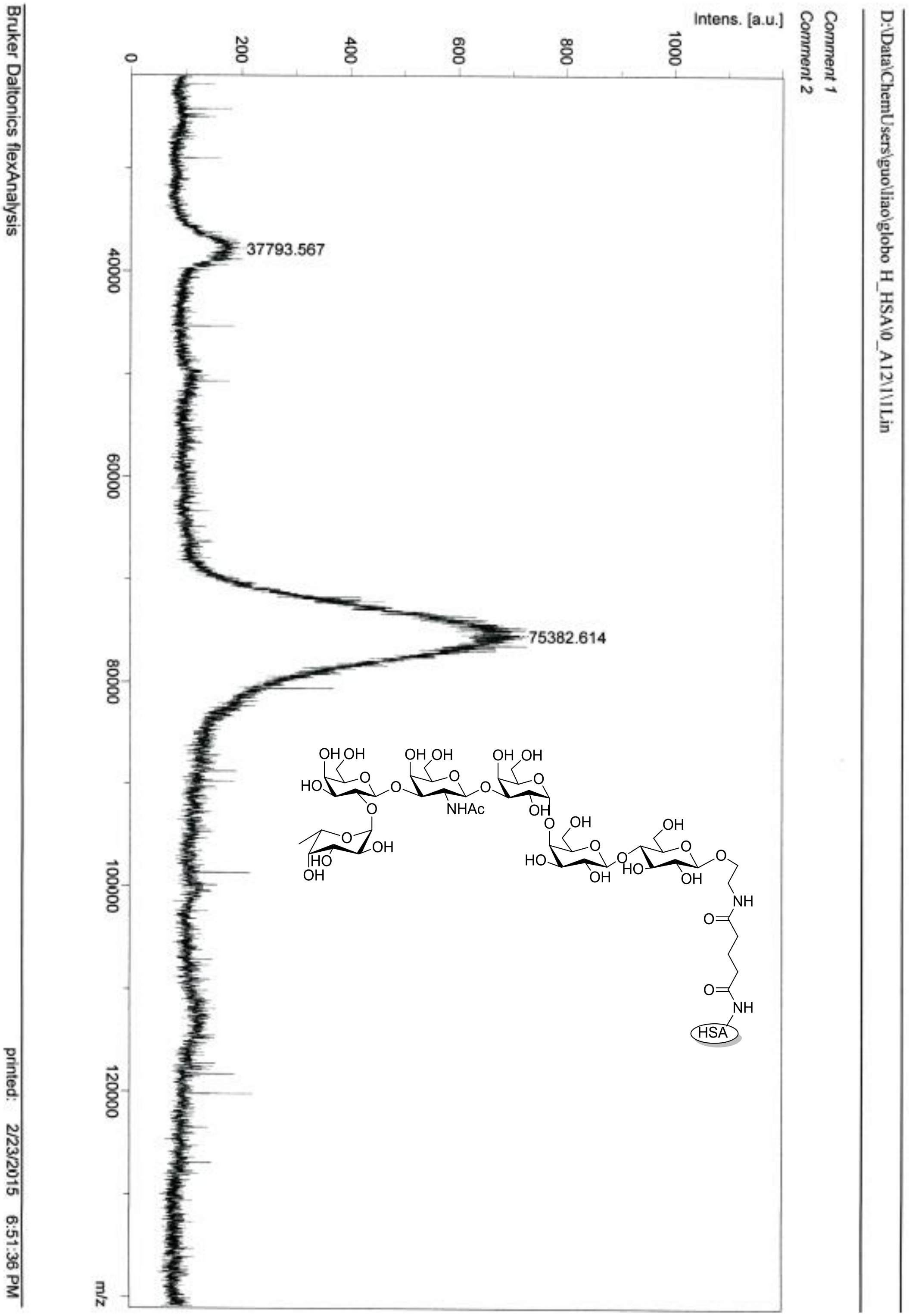

MALTI-TOF MS spectrum of compound **3**

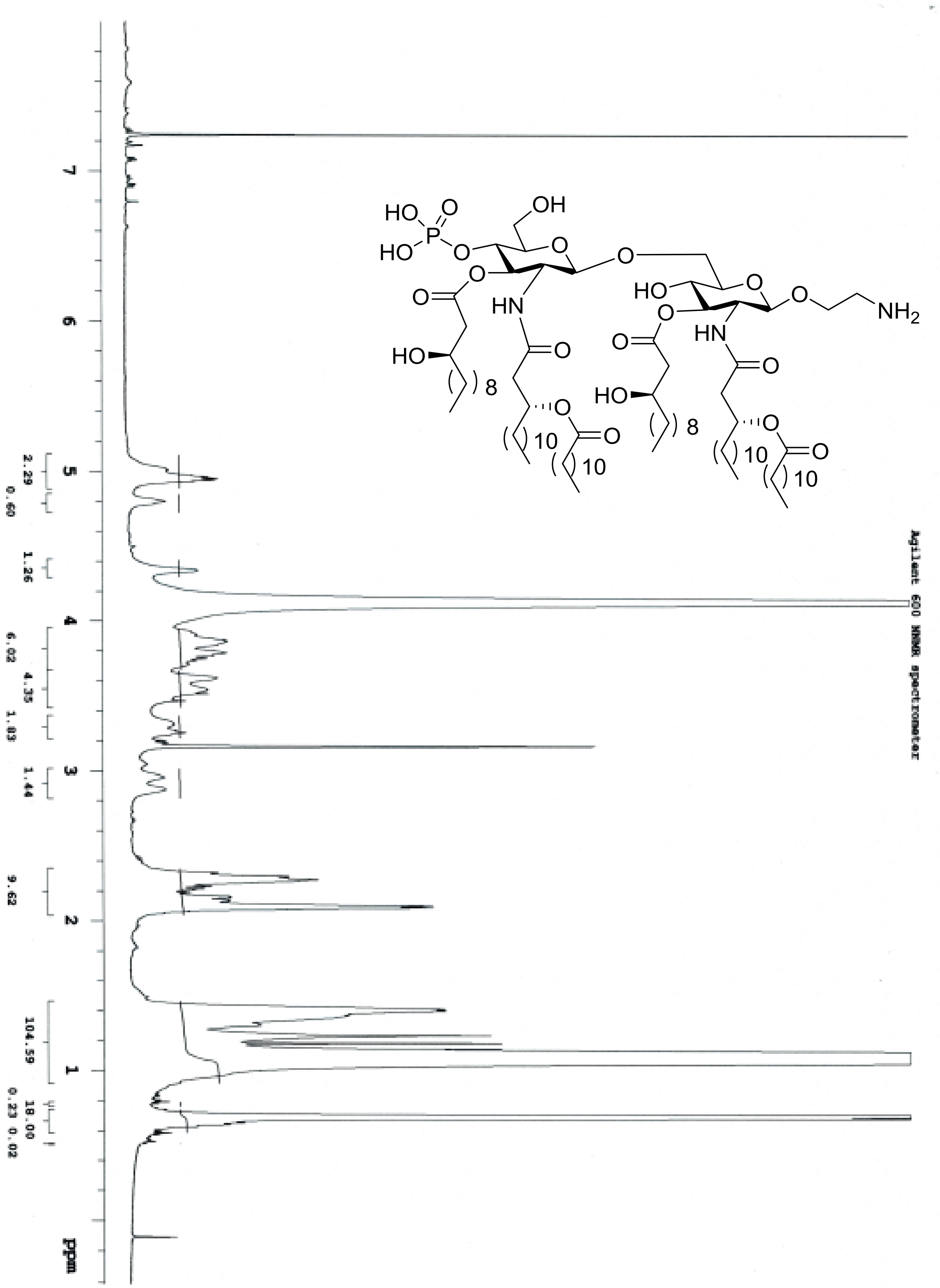

^{1}H NMR Spectrum of compound **S2** [$CDCl_3$:CD_3OD:D_2O (3:3:1) 600 MHz]

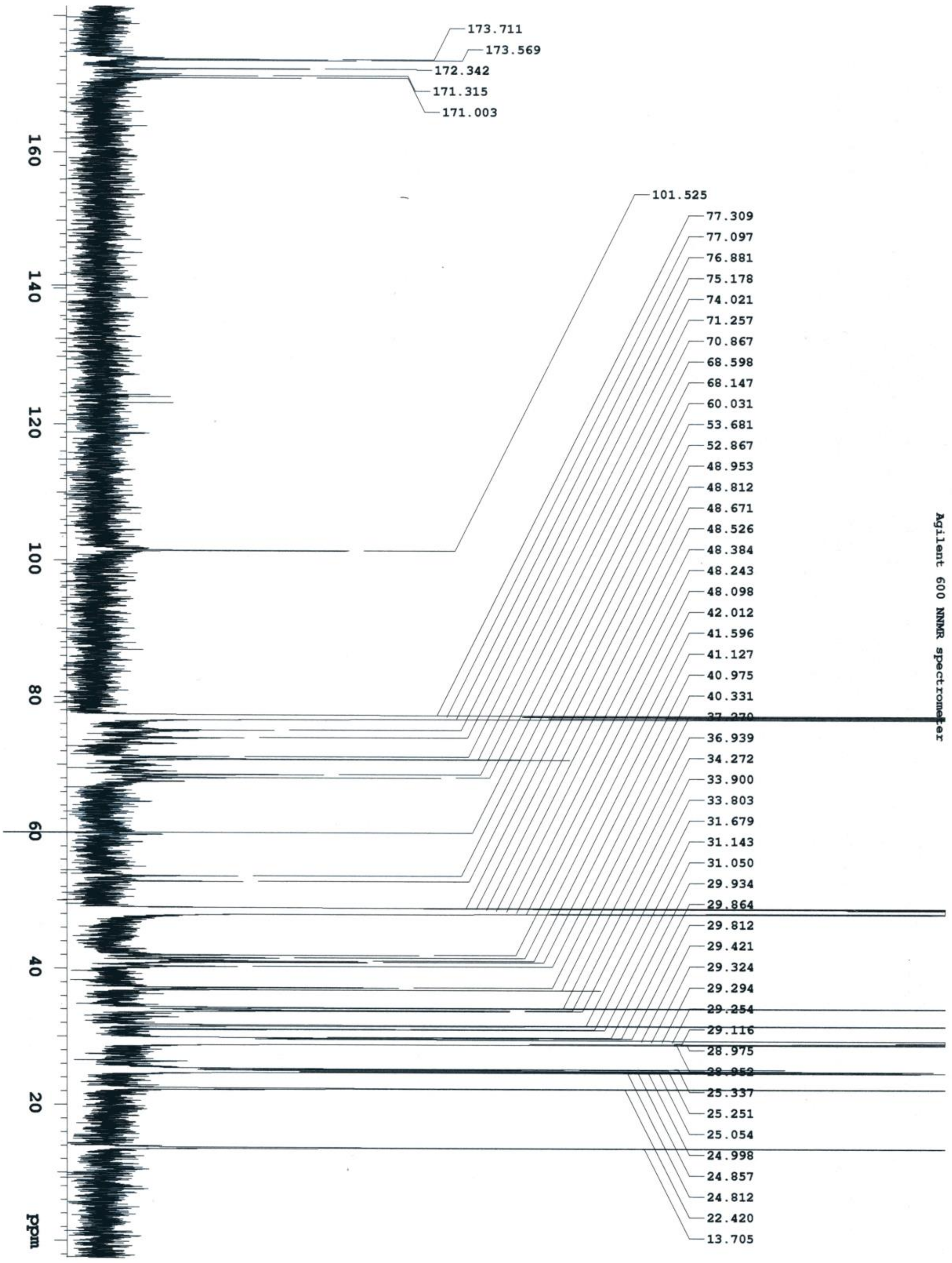

^{13}C NMR Spectrum of compound **S2** [$CDCl_3$:CD_3OD:D_2O (3:3:1) 600 MHz]

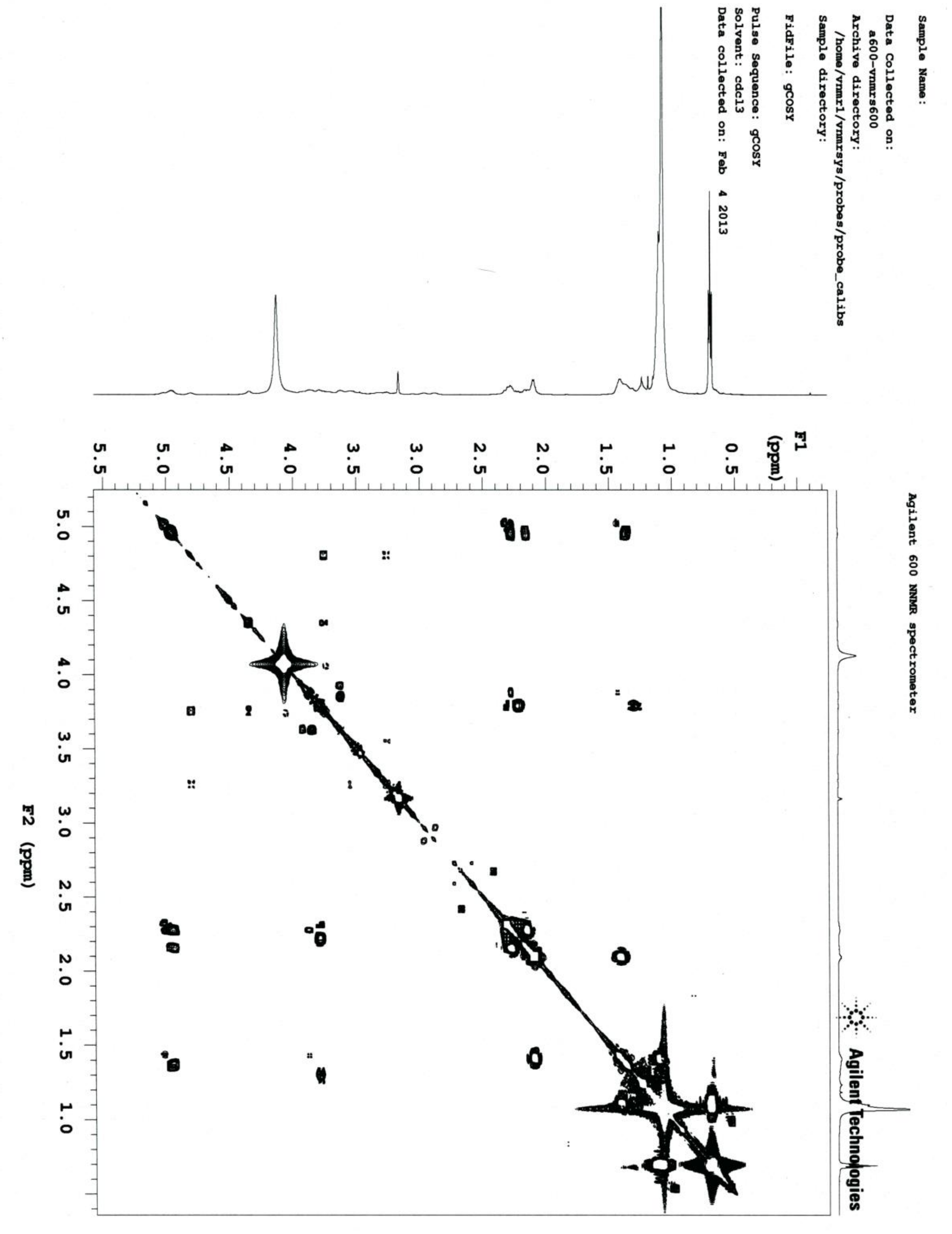

^{1}H-^{1}H COSY NMR Spectrum of compound **S2** [$CDCl_3$:CD_3OD:D_2O (3:3:1) 600 MHz]

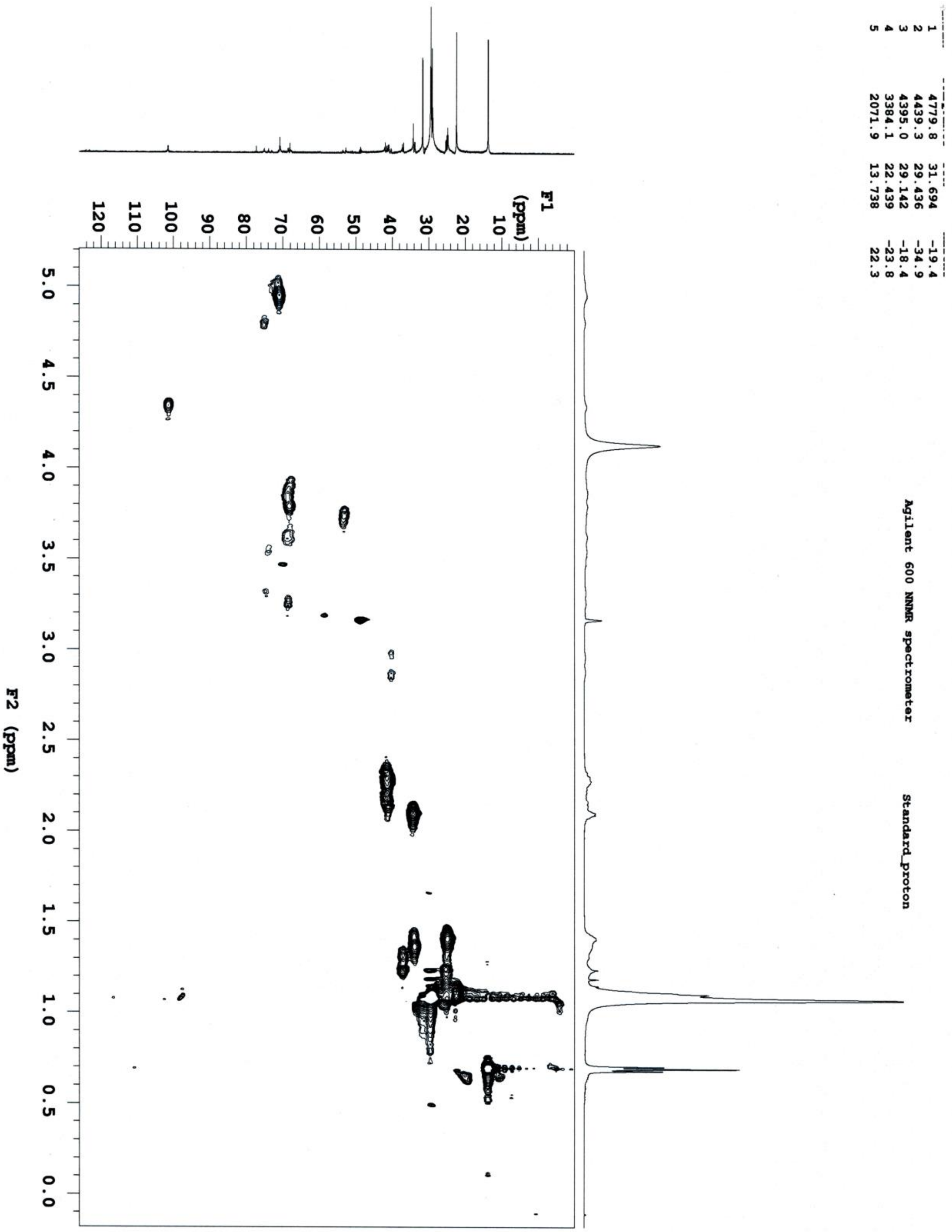

^{1}H-^{13}C HMQC NMR Spectrum of compound **S2** [$CDCl_3$:CD_3OD:D_2O (3:3:1) 600 MHz]

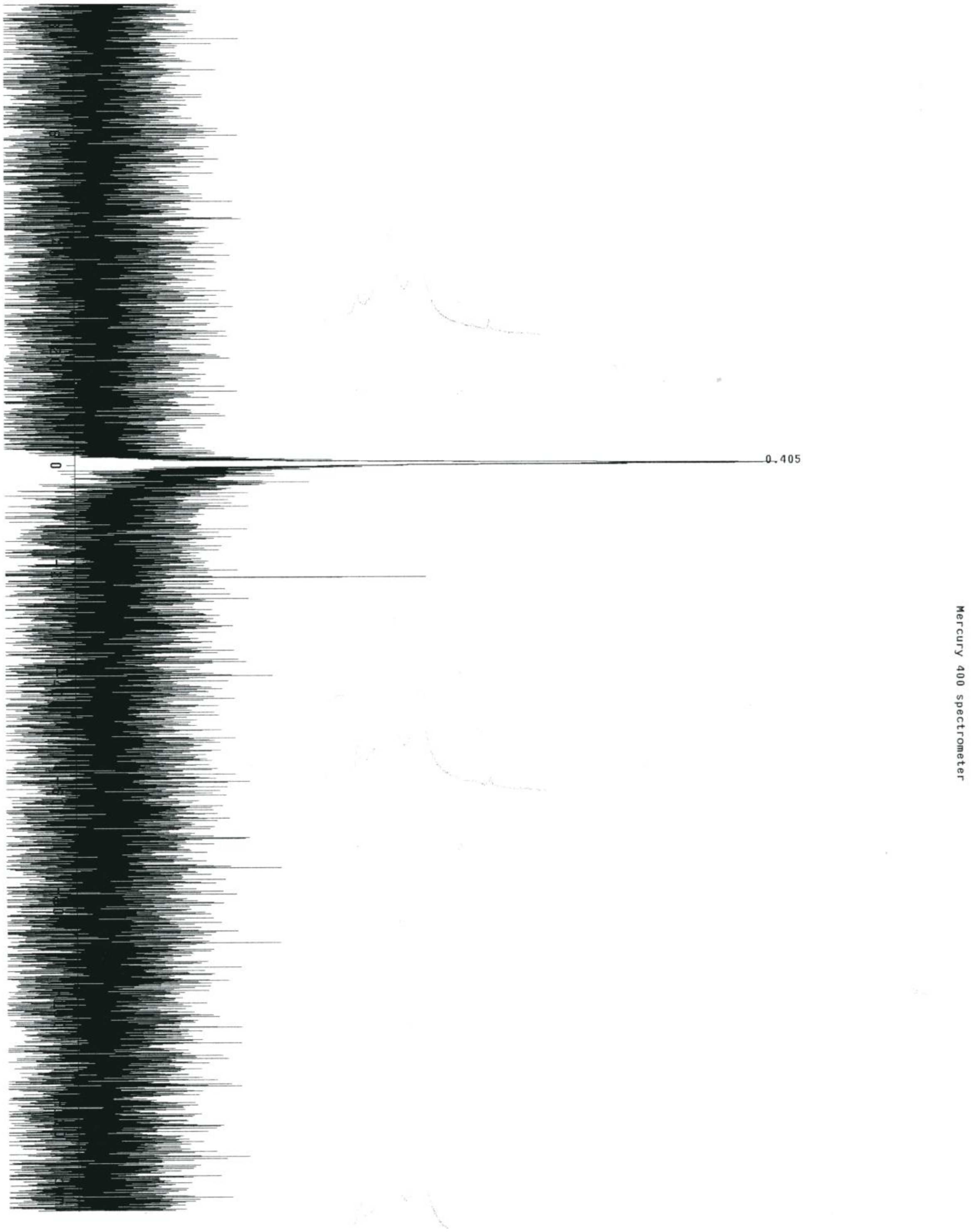

^{31}P NMR Spectrum of compound **S2** [$CDCl_3$:CD_3OD:D_2O (3:3:1) 400 MHz]

Elemental Composition Report **Page 1**

Single Mass Analysis
Tolerance = 5.0 PPM / DBE: min = -1.5, max = 100.0
Element prediction: Off
Number of isotope peaks used for i-FIT = 6

Monoisotopic Mass, Odd and Even Electron Ions
312 formula(e) evaluated with 2 results within limits (all results (up to 1000) for each mass)
Elements Used:
C: 90-90 H: 0-200 N: 0-5 O: 0-25 P: 0-1
G.Liao liao-3-126 inMeOH+DCM+Water Cone(V)60 LCT Premier XE KD128 14:28:36 23-Mar-2015
1503023_51 1103 (20.378) Cm (1099:1234-193:296x2.000) TOF MS ES+
3.83e+003

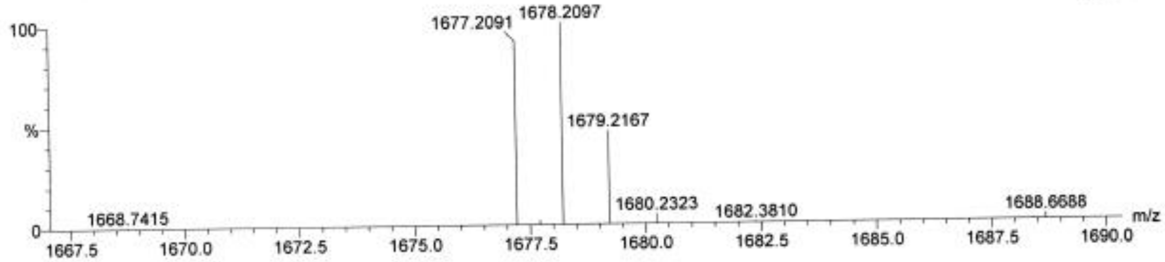

Minimum:				-1.5			
Maximum:		3.0	5.0	100.0			
Mass	Calc. Mass	mDa	PPM	DBE	i-FIT	i-FIT (Norm)	Formula
1677.2091	1677.2092	-0.1	-0.1	7.5	54.1	0.1	C90 H171 N3 O22 P
	1677.2174	-8.3	-4.9	7.5	56.9	2.8	C90 H170 N3 O24

HRMS (ESI MS) spectrum of compound **S2**
